# Supplementary material for: Mitochondrial position responds to glucose stimulation in a model of the pancreatic beta cell
Source: Biophys J. 2025 Dec 18;125(1):168–76. doi: 10.1016/j.bpj.2025.11.018 (PMC12821015; doi:10.1016/j.bpj.2025.11.018)
Supplement: Document S1. Figures S1–S12 [file mmc1.pdf]

Biophysical Journal, Volume *125*

Supplemental information

Mitochondrial position responds to glucose stimulation in a model of the pancreatic beta cell

Luis Perez, Xue Wen Ng, Michael Mohs, David W. Piston, and Shankar Mukherji

Supplementary Figures

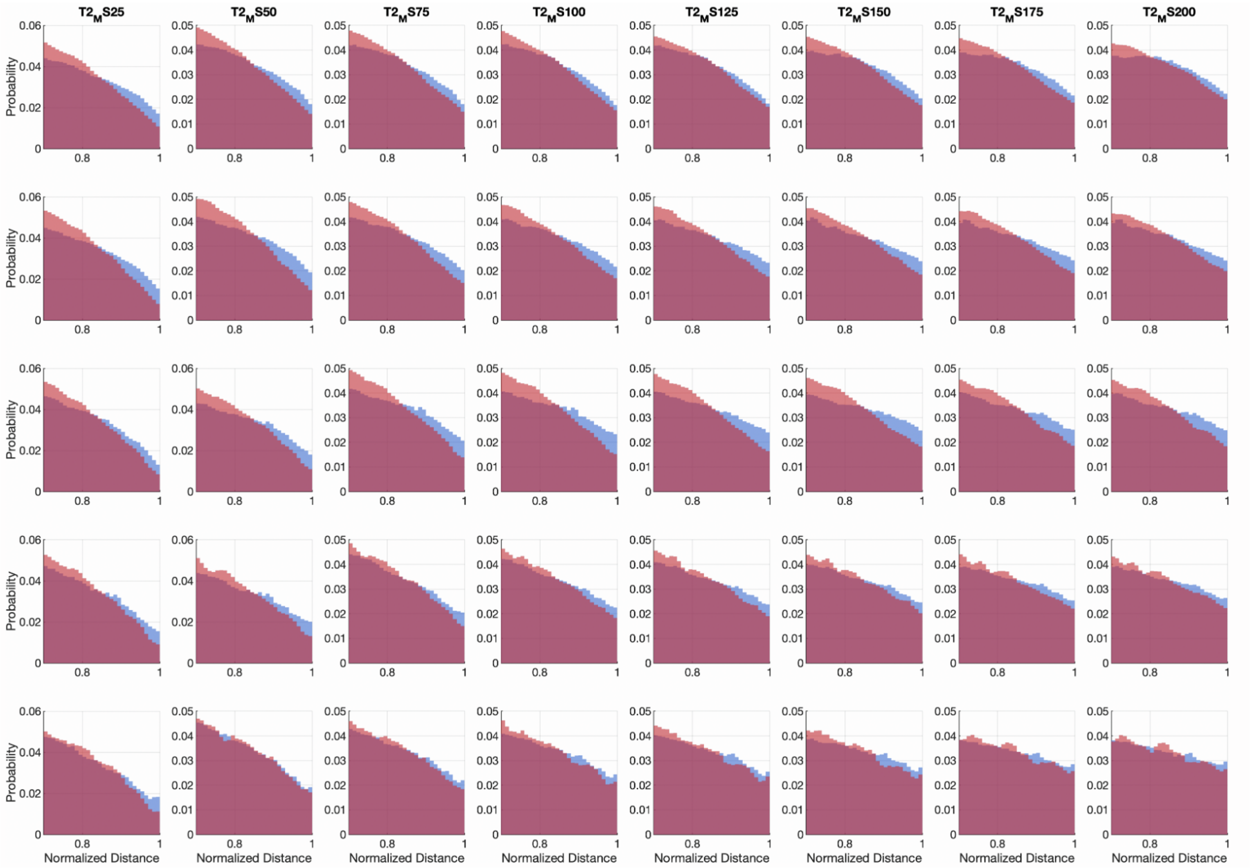

Figure S1. Sensitivity analysis of mitochondrial pixel probability distributions as a function of smoothing and thresholding parameters. Connected component size thresholding parameters are varied across columns between 25 to 200 pixels, fluorescence intensity thresholding parameters are varied across rows from 100-1000.

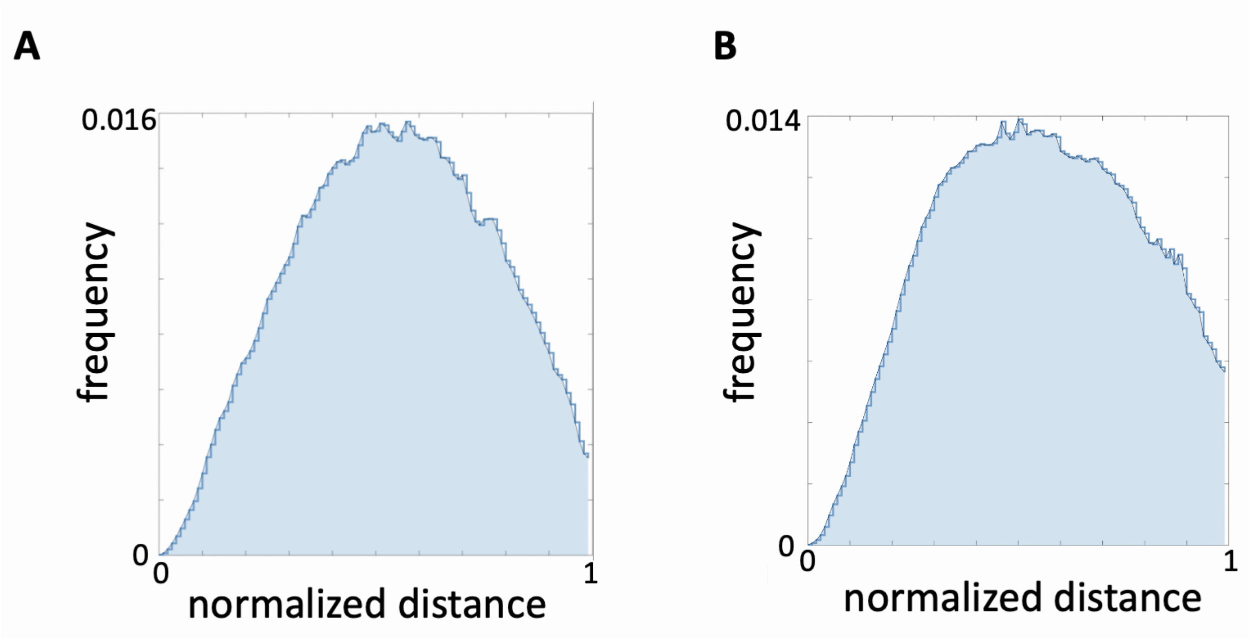

Figure S2: Mitochondrial pixel normalized radial density distributions for cells stimulated with A) 2mM glucose and B) 25mM glucose.

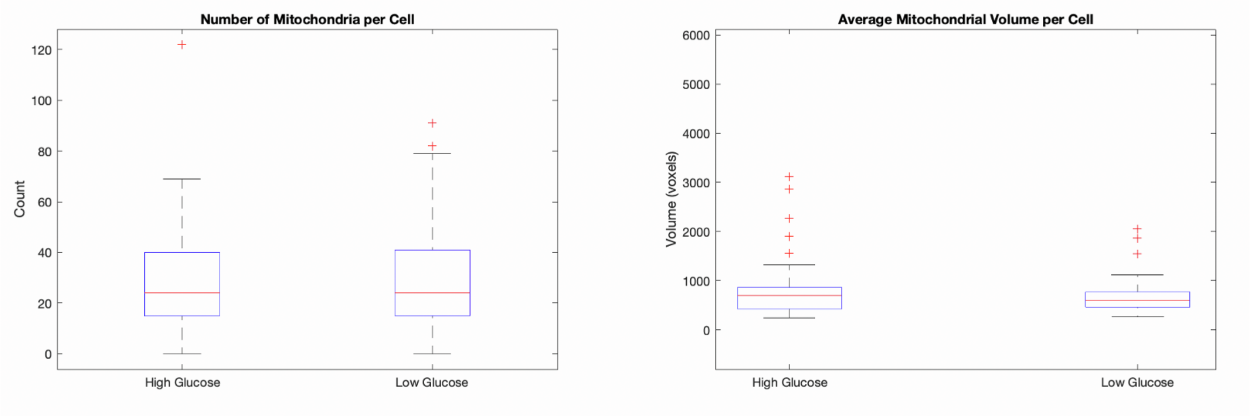

Figure S3. Box and whisker plots showing the median (red line) and 50% interquartile range (blue boxes) of number of mitochondria per cell and average mitochondrial volume per cell for cells cultured in 25mM glucose (high glucose) and 2mM glucose (low glucose)

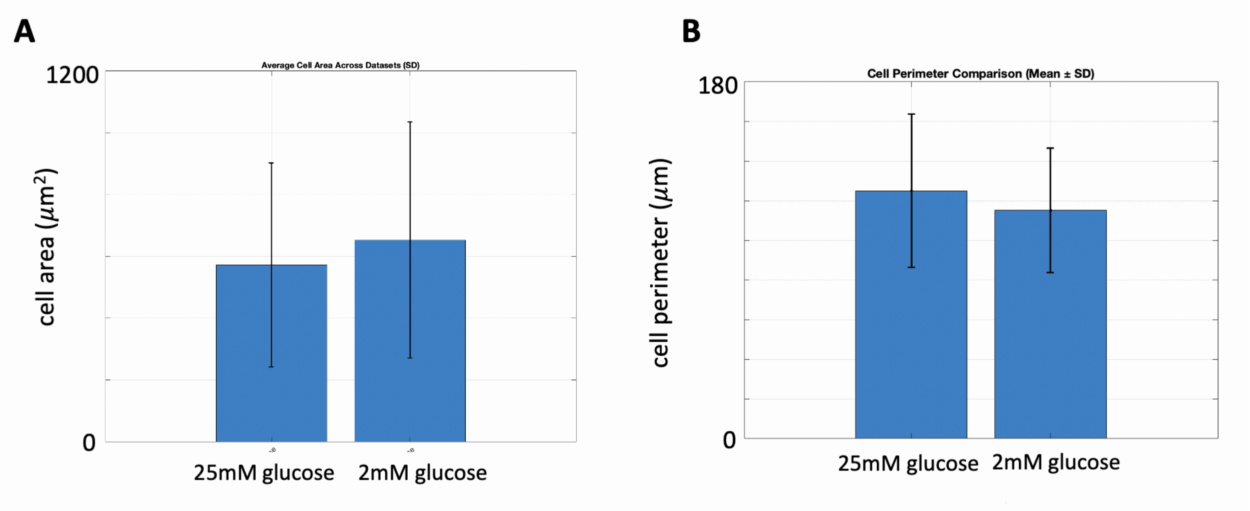

Figure S4: Comparison of cell area (A; p-value = 0.1882, t-test) and cell perimeter (B; p-value = 0.2114, t-test) for cells stimulated with 25mM and 2mM glucose.

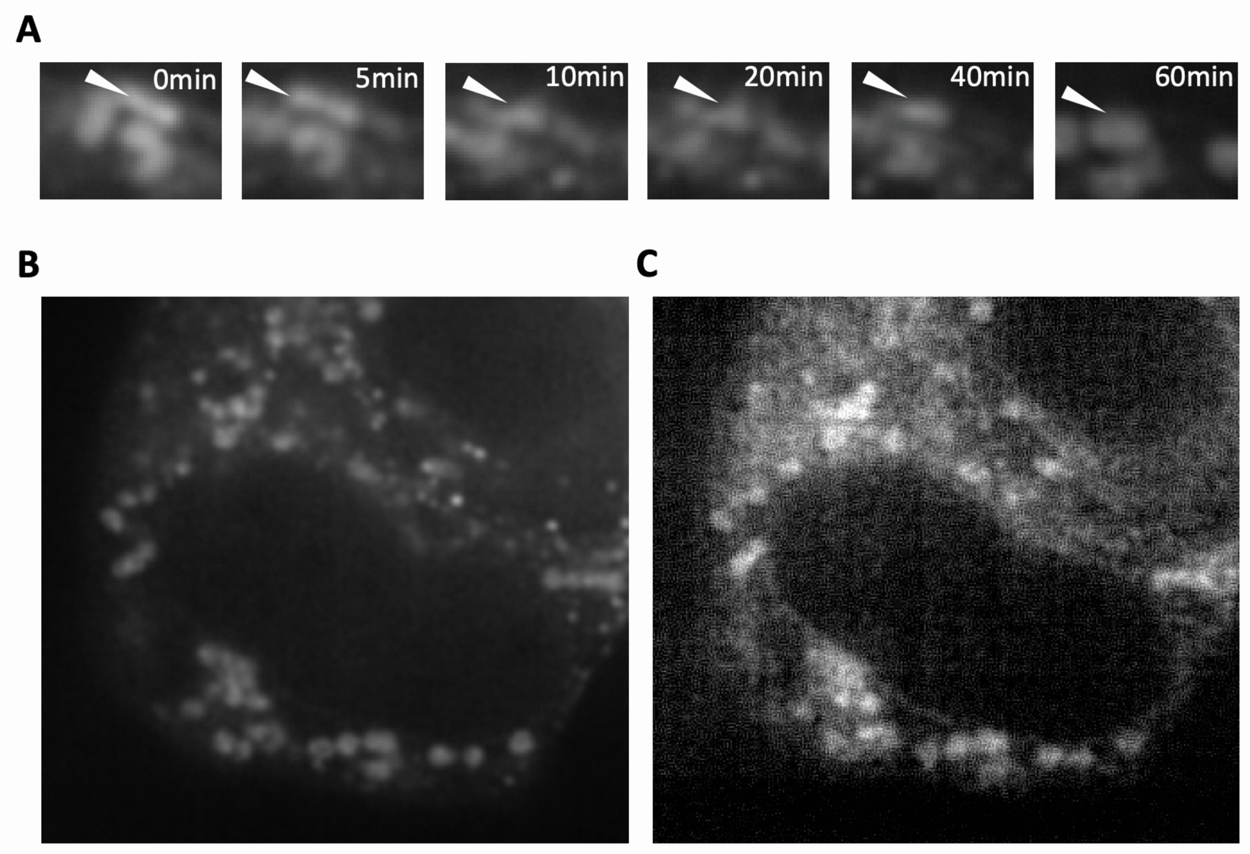

Figure S5: Effect of FCCP on MitoTrackerCMXRos signal. A) Timelapse imaging of single mitochondrion labeled with MitoTrackerCMXRos over a course of 60min. Comparison of fluorescence signal of mitochondrial dually labeled with MitoTrackerCMXRos (B) and MitoTracker Green FM (C).

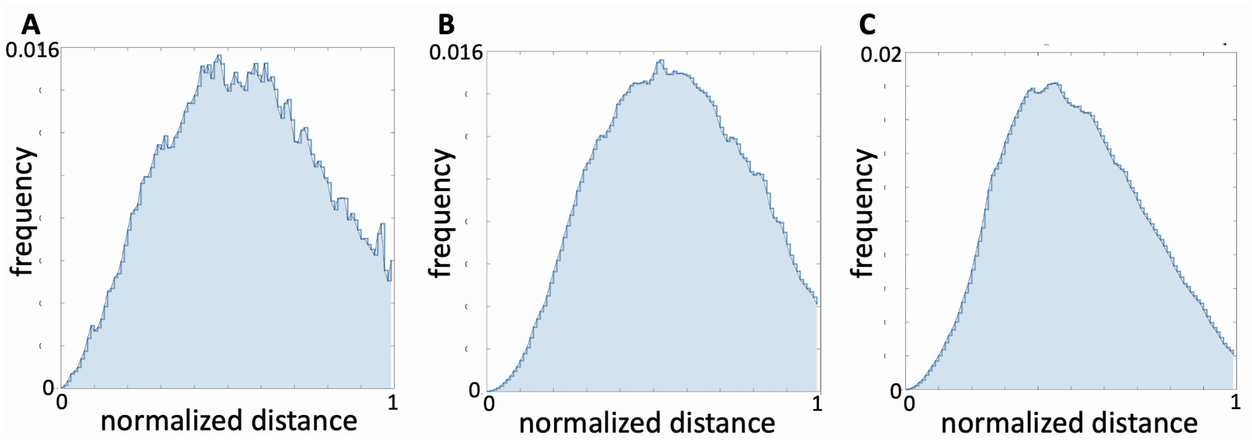

Figure S6: Mitochondrial pixel normalized radial distance density distributions for cells treated with: A) FCCP, B) nocodazole, and C) somatostatin.

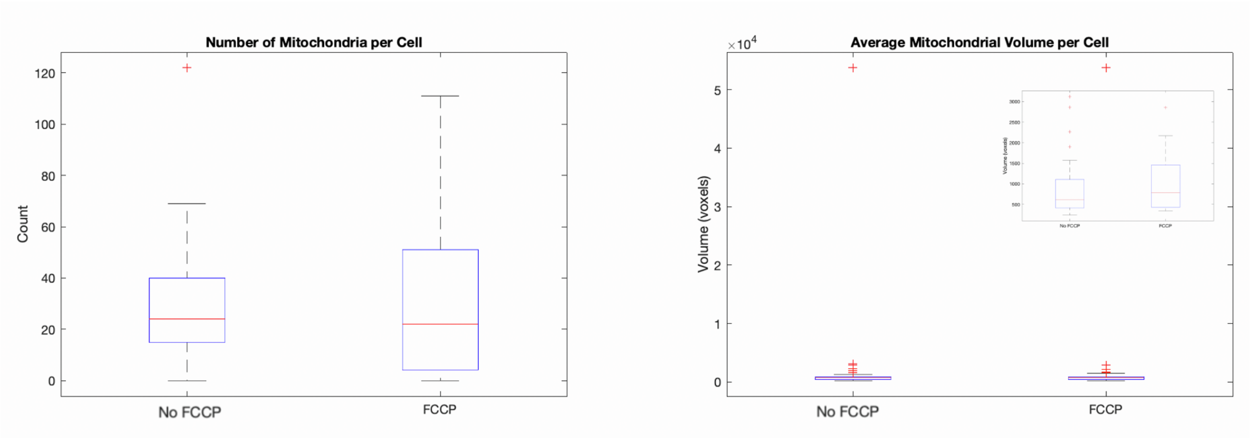

Figure S7. Box and whisker plots showing the median (red line) and 50% interquartile range (blue boxes) of number of mitochondria per cell and average mitochondrial volume per cell in cells cultured in the absence and presence of the uncoupling agent FCCP. Inset in right panel shows box and whisker plots with points with outlier average mitochondrial volume datapoints removed to facilitate comparison between the two populations.

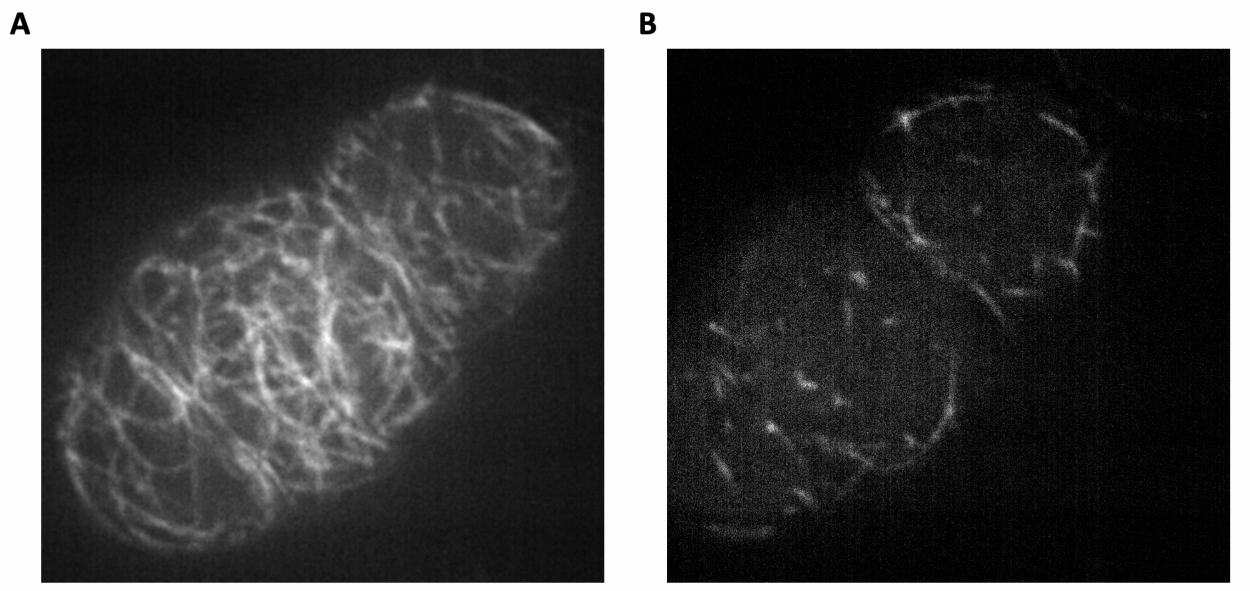

Figure S8: Microtubule networks visualized with Spy650-Tubulin: A) prior to nocodazole treatment, B) following nocodazole treatment.

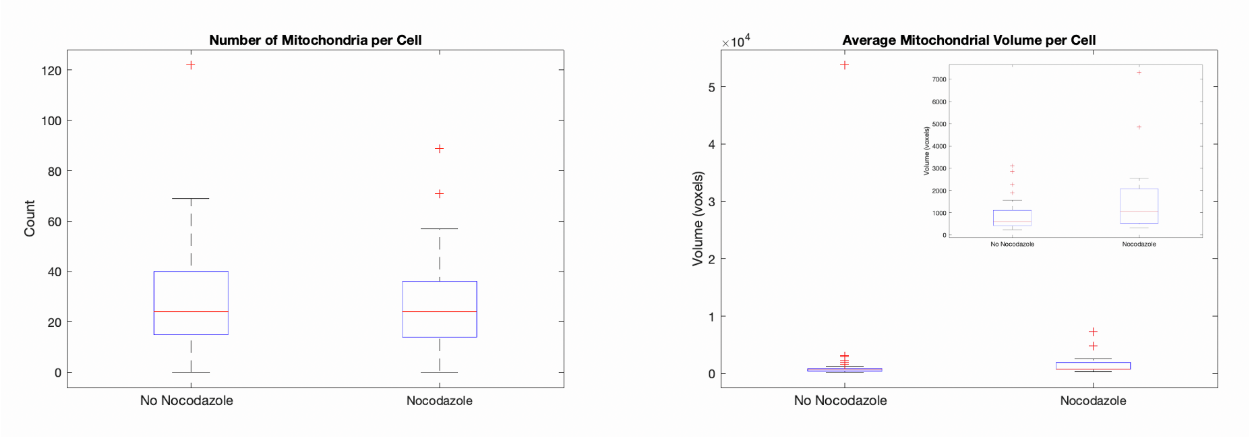

Figure S9. Box and whisker plots showing the median (red line) and 50% interquartile range (blue boxes) of number of mitochondria per cell and average mitochondrial volume per cell in cells cultured in the absence and presence of the microtubule disrupting agent nocodazole. Inset in right panel shows box and whisker plots with points with outlier average mitochondrial volume datapoints removed to facilitate comparison between the two populations.

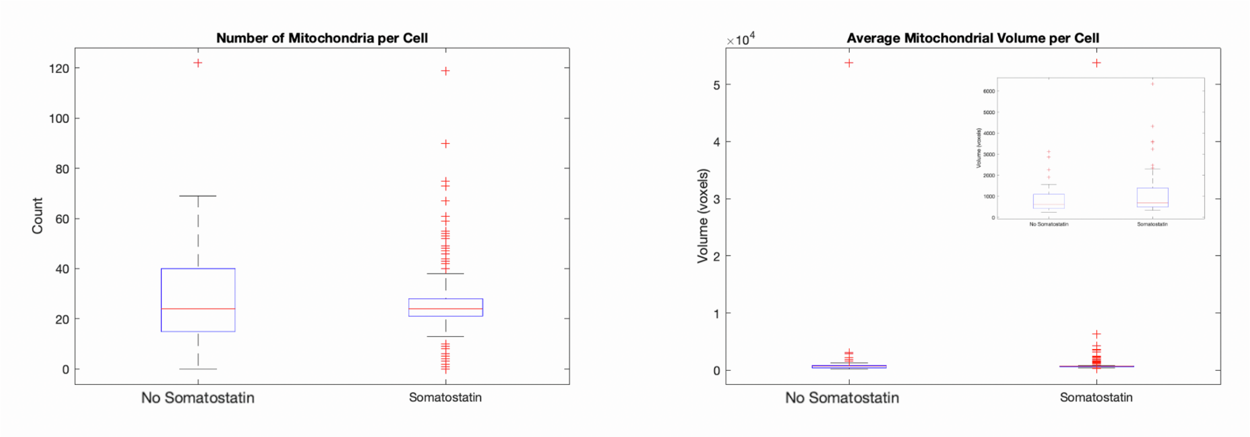

Figure S10. Box and whisker plots showing the median (red line) and 50% interquartile range (blue boxes) of number of mitochondria per cell and average mitochondrial volume per cell in cells cultured in the absence and presence of somatostatin. Inset in right panel shows box and whisker plots with points with outlier average mitochondrial volume datapoints removed to facilitate comparison between the two populations.

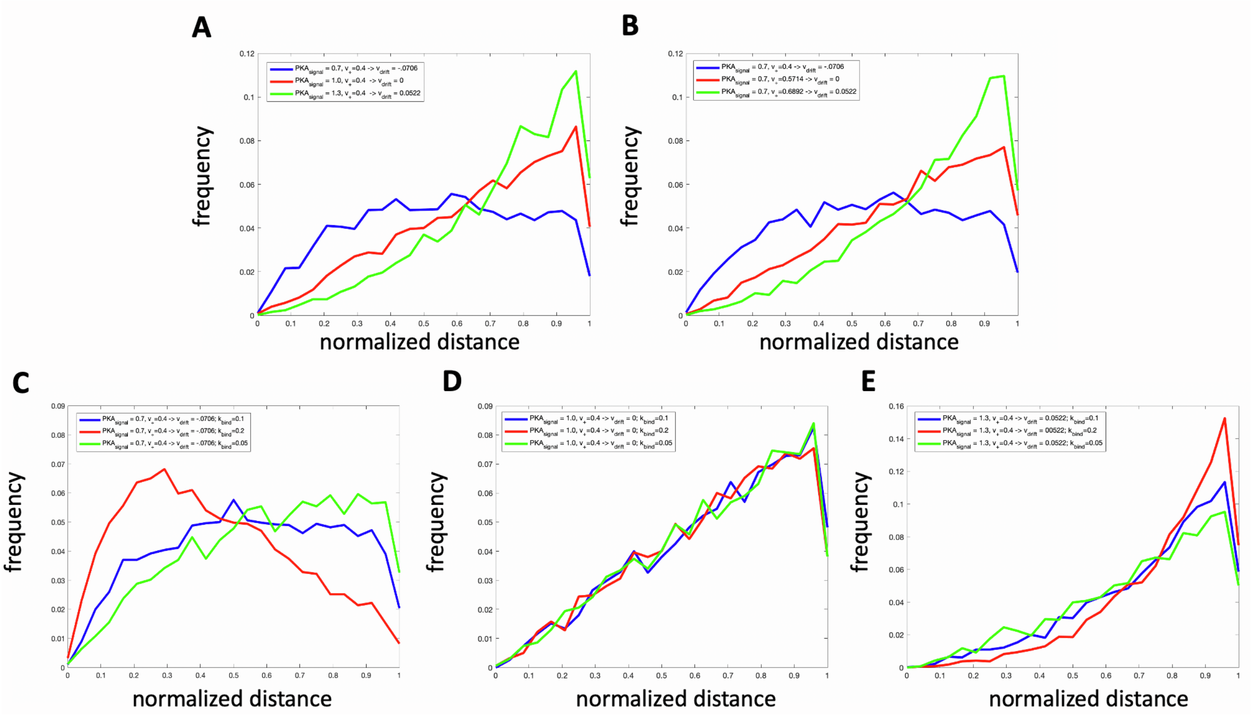

Figure S11: Model parameter sensitivity analysis as plotted in mitochondrial normalized radial distance distributions from simulations. A) Results of sensitivity analysis changing v_{drift} by changing PKA_{signal} but holding v_+ constant. B) Results of sensitivity analysis changing v_{drift} by changing v_+ but holding PKA_{signal} constant. C) Results of sensitivity analysis changing k_{bind} and keeping $v_{drift} < 0$ holding v_+ and PKA_{signal} constant. D) Results of sensitivity analysis changing k_{bind} and keeping $v_{drift} = 0$ holding v_+ and PKA_{signal} constant. E) Results of sensitivity analysis changing k_{bind} and keeping $v_{drift} > 0$ holding v_+ and PKA_{signal} constant.

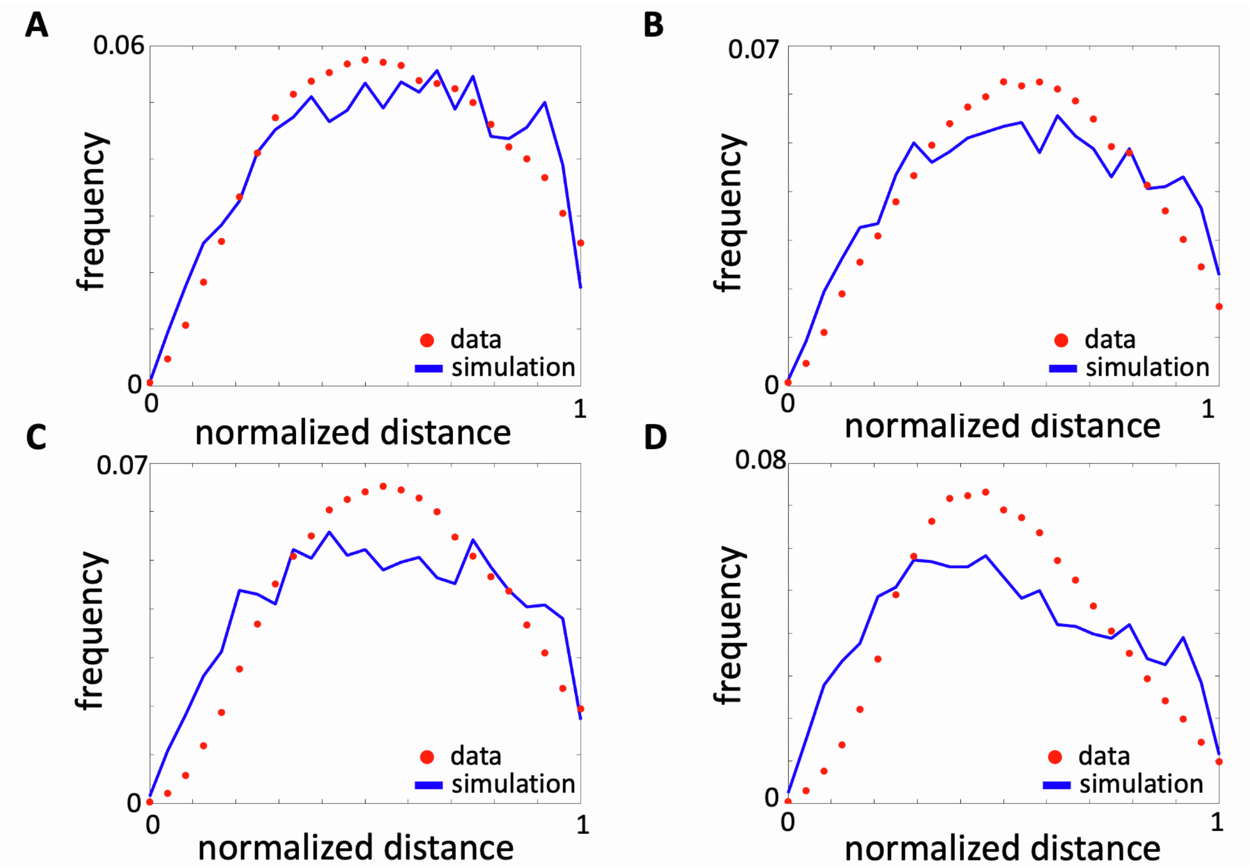

Figure S12: Comparison of random walk model of mitochondria to experimental data of mitochondrial pixel normalized distance distribution. A) 25mM glucose (parameter values k_{bind} = 0.1, PKA_{signal} = 0.7, v_+ = 0.4, v_- = 0.4, step size = 0.2), B) 2mM glucose (parameter values k_{bind} = 0.05, PKA_{signal} = 0.64, v_+ = 0.4, v_- = 0.4, step size = 0.15), C) nocodazole (parameter values k_{bind} = 0.05, PKA_{signal} = 0.7, v_+ = 0.2, v_- = 0.4, step size = 0.2), D) somatostatin. (parameter values k_{bind} = 0.1, PKA_{signal} = 0.6, v_+ = 0.4, v_- = 0.4, step size = 0.2)
