# Supplementary material for: Mitochondrial position responds to glucose stimulation in a model of the pancreatic beta cell
Source: Biophys J. 2025 Dec 18;125(1):168–76. doi: 10.1016/j.bpj.2025.11.018 (PMC12821015; doi:10.1016/j.bpj.2025.11.018)
Supplement: Document S2. Article plus supporting material [file mmc2.pdf]

Biophysical Journal
Article

Biophysical Society

Mitochondrial position responds to glucose stimulation in a model of the pancreatic beta cell

Luis Perez,[1] Xue Wen Ng,[2] Michael Mohs,[1] David W. Piston,[2] and Shankar Mukherji[1,2,*]
[1]Department of Physics, Washington University in St. Louis, Saint Louis, Missouri and [2]Department of Cell Biology & Physiology, Washington University in St. Louis, Saint Louis, Missouri

ABSTRACT The compartmentalization of eukaryotic cells into membrane-bound organelles with specific subcellular positioning enables precise spatial and temporal control of cellular functions. Although functionally significant mitochondrial localization has been demonstrated in cells such as neurons, it remains unclear how general these cell principles are. Here, we examine the spatial organization of mitochondria within MIN6 pancreatic beta cells under variable glucose conditions. We observe glucose-dependent redistributions of mitochondria, favoring peripheral localization at elevated glucose levels. Our results, formalized into a stochastic model of mitochondrial trafficking, suggest that active mitochondrial transport along microtubules and PKA signaling activity, but not ATP synthesis, are critical regulators of this redistribution. These results suggest that environmentally responsive mitochondrial subcellular positioning may represent a general regulatory mechanism in even non-polarized cell types.

SIGNIFICANCE It is widely believed that subcellular positioning of organelles plays important roles in their function within the context of the cell. This phenomenon is best illustrated in mitochondrial positioning in highly polarized cells such as neurons. Here, we show that this concept may also apply to less polarized cells that nevertheless have spatially structured flows of metabolites across different parts of the cell by measuring mitochondrial spatial distributions in a pancreatic beta cell-like model system. Using quantitative imaging and mathematical modeling, we suggest glucose stimulation, by acting through PKA-regulated anterograde transport of mitochondria along microtubules, shifts mitochondrial density toward the cell periphery. Spatial redistribution of mitochondria could reflect their role in connecting sensed levels of glucose to output insulin levels.

INTRODUCTION

The organization of eukaryotic cells into organelles allows for spatiotemporal organization of biological processes, as seen via the specialized functionality of each respective organelle. Organelles are distributed throughout the cell in membrane-bound compartments and allow the cell to spatially segregate specific biochemical processes. Mitochondria, among the most dynamic organelles, are primarily responsible for generating energy in most eukaryotic cells. Elucidating how the biophysical context of mitochondria, in terms of both the morphology of the mitochondrion itself as well as its interactions with the rest of the cell, regulates their cell biological functions remains a frontier area of investigation. Prior studies have mostly focused on mitochondrial morphology. Mitochondria behave like a constantly evolving network within the cell: the network can expand by undergoing fusion to other mitochondria and is broken down via fission or mitophagy in the case of damaged material (1–5). The number, size, and genetic material (mtDNA) of individual mitochondrial structures have a well-established connection to the metabolic function of mitochondria and their ability to produce ATP (6–10). These analyses, however, are more focused on properties of individual mitochondria with comparatively less emphasis on how they operate within the context of their host cells.

One potentially crucial, but less studied, factor coordinating mitochondrial activity and cellular function is the subcellular spatial positioning of mitochondria. Understanding how mitochondrial positioning responds to cellular demand may give us insight into how the cell optimizes organelle activity. Prior studies suggest that the cell appears to exert control over subcellular mitochondrial position. For example, it has been shown that cells express anchoring complexes that specifically exert control over mitochondrial positioning (11). Furthermore, studies from cardiomyocytes have documented a

Submitted July 3, 2025, and accepted for publication November 12, 2025.
*Correspondence: smukherji@wustl.edu
Editor: Guy Genin.
https://doi.org/10.1016/j.bpj.2025.11.018
© 2025 The Author(s). Published by Elsevier Inc. on behalf of Biophysical Society.
This is an open access article under the CC BY license (http://creativecommons.org/licenses/by/4.0/).

preferred spatial allocation of mitochondria biomass within the cell (12). Perhaps the most extensive observations of control over mitochondrial subcellular position have been shown in neurons, where both experimental and theoretical studies (13–16) have indicated that neuronal mitochondria are trafficked to and retained in neuronal segments with high metabolic demand, typically in distal axons. The inference drawn from these studies is that since mitochondria produce energy for the cell, they preferentially position where those energy demands are highest. Given the highly polarized nature of neurons, whose elongated and highly branched geometry would significantly inhibit the cell's ability to deliver adequate amounts of ATP by diffusion alone to distal axonal processes from the cell body, the need for active transport of mitochondria has a strong biophysical rationale. Whether this same logic can be applied to other, less polarized cell types is less examined and less clear.

One such cell type that exhibits relative morphological simplicity and strong demands of mitochondrial function is the pancreatic beta cell. In particular, beta cells engage in a highly specialized form of mitochondrial usage in which ATP production via oxidative phosphorylation not only provides energy for the cell but also essentially acts as a sensor of blood glucose levels to drive subsequent insulin secretion. Numerous aspects of glucose-stimulated insulin secretion (GSIS) exhibit significant heterogeneity in the cell. GSIS begins with the import of glucose into beta cells (17) at passive transporters at the cell membrane. Looked at as a sensor, mitochondrial activity then converts the glucose signal into a change in the cytosolic ATP/ADP ratio, which results in closing of membrane K_{ATP} channels, which leads to membrane depolarization and calcium influx (18) into the cytosol. This calcium influx ultimately drives exocytosis of insulin granules whose positions are not random throughout the cell (19). The function of beta cell mitochondria thus depends on interactions heterogeneously distributed throughout the space of the cell. The known link between glucose stimulation, and subsequent calcium influx leading to PKA signaling, and PKA signaling having the capacity to drive kinesin-based anterograde mitochondrial transport (20) motivates the importance of experimentally characterizing mitochondrial subcellular positioning.

Here, we show that MIN6 cells show a subtle but significant shift in the spatial distribution of their mitochondria depending on their local glucose environment. Our results suggest that organelle positioning is an important factor of mitochondrial functionality even in the context of morphologically simple cells.

MATERIALS AND METHODS

Cell culture

MIN6 cells were grown in Dulbecco's Modified Eagle's Medium (DMEM, 25 mmol/L glucose) equilibrated with 5% CO_2 and 95% air at 37°C. The medium was supplemented with 15% fetal bovine serum and 5% penicillin and streptomycin. MIN6 cells used in this study were harvested at passages 30–40.

TABLE 1 Imaging parameters used in MIN6 acquisition and analysis

Pixel size	0.0542 μm × 0.0542 μm
Z-step	0.2 μm
exposure time	488 nm, 200 ms
	640 nm, 500 ms
Gaussian sigma	2.0

Analysis of mitochondrial properties

Semiconfluent cells were grown on a separate dish in anticipation for imaging. On the day of the experiment, cells in their respective dishes were incubated with 100 nanomolar MitoTracker Red CMXRos for 1 h before imaging, adding the dye to a fresh aliquot of regular medium that the cells were grown in. After 1 h, the cells were rinsed with KRBH, which behaved as a clear imaging media for the cells.

Imaging protocol

Cells were imaged using a Nikon spinning disk confocal microscope, fully equipped with an incubator (Tokai-Hit) enabling full temperature, CO_2, and humidity control. Cells were grown in glass-bottom dishes, which were fixed on a microscope stage, and images were obtained using a PlanApo 100× 1.45 NA oil-immersion objective. Z stacks were obtained with a z spacing of 0.2 μm.

The cells were maintained at 37°C and 5% CO_2. The Red CMXRos dye was excited with a 561-nM laser, and fluorescence emission was detected through a 665-nM filter (red emission channel). Unless otherwise stated, gain levels, confocal aperture size, and laser power were adjusted to not saturate the detector but remained constant throughout the time-series of image acquisition.

Relevant imaging parameters are shown in Table 1.

Image analysis

Images were opened up using Fiji, where the entire field of view of cells was visible. We identified a cell that only minimally overlapped with a neighboring cell and then cropped this cell. This cropped cell was analyzed using a custom MATLAB script that identifies all mitochondrial pixels within the cell. Mitochondrial pixel identification begins with applying a 2D Gaussian smoothing filter to each Z slice of the fluorescence image data to denoise the image data (using a smoothing radius $\sigma = 2.0$) followed by a Laplacian filter to sharpen the image. After this preprocessing step, we create a binary mask of pixels whose fluorescence value is above a fixed threshold value (750 for our setup) for each Z slice of the fluorescence image. We then use the binary mask to identify mitochondria as 3D connected components defined using only pixels, whose 26 neighboring pixels in 3D are also included in the mask using the MATLAB "bwconncomp" command. We then filter the connected components to remove components that are too small (125 pixels or less) or too large (100,000 pixels or more) to represent mitochondria. We define the number of mitochondria in a cell to be the number of connected components in the cell obtained from the filtered connected component analysis and the average size of the mitochondria in a cell to be the average number of pixels across the connected components within a cell. These results are reported in Figs. S2–S5. The mitochondrial pixels used in the distance analysis belong to these filtered connected components identified from the fluorescence images.

To carry out the distance measurements, we begin by manually setting the center of the nucleus as the origin for each respective cell. For a given cell, for each identified mitochondrial pixel, we compute the Euclidean distance to the center of the nucleus. We divide this Euclidean distance by the maximum

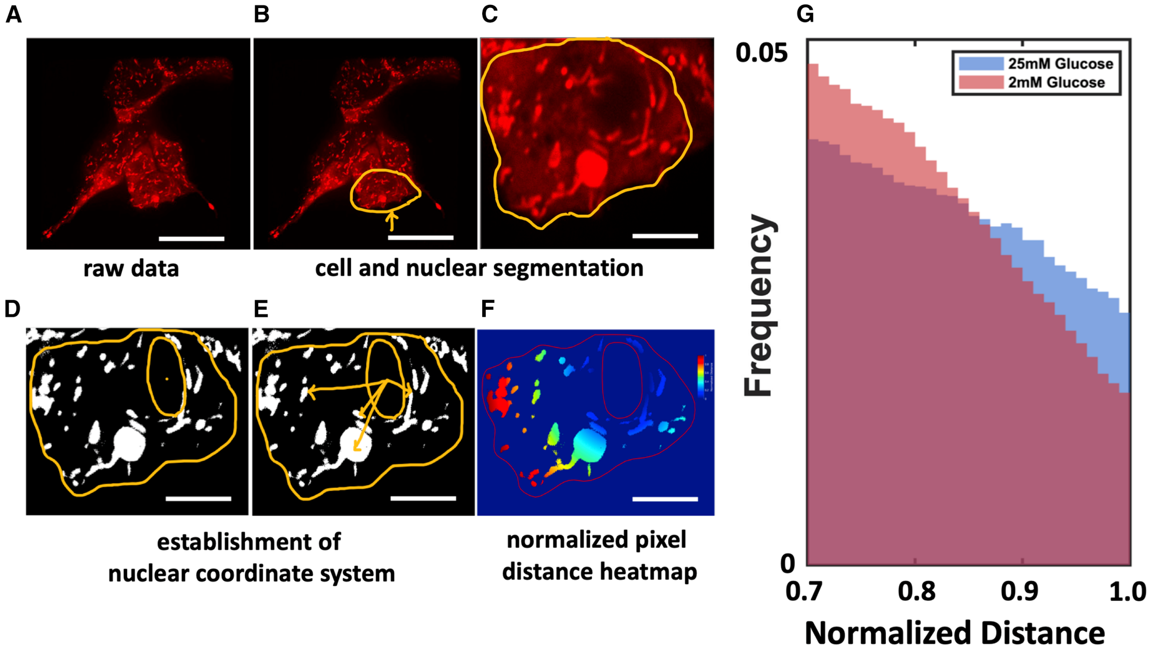

FIGURE 1 Mitochondrial spatial distribution in MIN6 cells under high and low glucose stimulation. Sample micrographs and schematic image analysis strategy of MitoTracker CMXRos-labeled MIN6 cells: (*A*) in the first subpanel, we show a sample image of our raw image data; (*B* and *C*) an individual cell is identified (*yellow border*); (*D*) the nucleus is manually identified as a region of low fluorescence intensity; (*E*) distances from the center of the nucleus to various mitochondrial pixels are indicated in the schematic; and (*F*) we display a heatmap depicting the distances of each mitochondrial pixel to the center of the nucleus. In (*A*) and (*B*), the scale bar represents 20 μm. In (*C*)–(*F*), the scale bar represents 10μm. (*G*) Distribution of mitochondrial pixel distances from the center of the nucleus for the 30% most distant pixels from cells stimulated with 25 mM (*red histogram*; $N = 85$ cells) and 2 mM (*blue histogram*, $N = 66$ cells) glucose; the two distributions are statistically distinguishable (Kolmogorov-Smirnov test, $p < 1.9 \times 10^{-3}$).

pixel distance in the given cell under analysis to obtain the normalized distance. We then pool all normalized radial distances for each pixel from all cells in a given condition to construct the histograms shown in Figs. 1 and 2.

Titration experiments

On the day before the experiment, MIN6 cells from a T-25 flask were monitored to verify the approximate optimal confluency for the cell line at 70%–80% density. We incubated the cells with Trypsin-EDTA (1×) for 15 min, or until the cell layer was dispersed, to allow for proper aliquoting, with a similar optimal density profile, to culture vessels suitable for imaging. For static incubation, the cells were incubated in their normal media as outlined above. On the day of the experiment, cells were preincubated in the respective perturbation conditions: 2mM glucose, 10 nM FCCP, 10 μM somatostatin, and 10 μM nocodazole for 60 min. We chose 60-min stimulation times to ensure that mitochondria had sufficient time to achieve a new steady-state spatial distribution following stimulation, given that mitochondria are transported along microtubules on the scale of μm/s but that glucose stimulation still results in insulin secretion at that timepoint. Given the use of 60 min of stimulation time for glucose, we repeated this same stimulation time for FCCP, nocodazole, and somatostatin for consistency. Just before imaging, the culture medium was rinsed and replaced with the appropriate imaging medium (1× KRBH). This was supplemented with the respective conditions of the titration to ensure maintenance of cellular environment during live imaging. Cells in the dish were then placed in the incubator and underwent live-cell imaging in 5% CO_2 conditions at 37°C.

Mitochondrial biased random walk simulation

The mitochondrial random walk simulation was carried out in MATLAB. In brief, mitochondria could exist in one of two states: either bound to microtubules or not, and they performed motion within a circular disk region with fixed radius 1. The initial position of a simulated mitochondrion was drawn from a uniform distribution across the disk. The unbinding rate constant k_{unbind} was set to 1, thus effectively rescaling simulation time to the timescale of mitochondrial unbinding from microtubules. The simulation used discrete time steps. At each time step, a simulated mitochondrion was allowed to randomly switch microtubule binding states with probability proportional the binding and unbinding rate constants. For unbound mitochondria, the mitochondrion was allowed to take a random step in both X and Y directions corresponding approximately to a diffusion constant on the order of 1 $\mu m^2/s$; if the random step would take the mitochondrion outside the circular disk region, then reflecting boundary conditions were applied to confine the mitochondrion to the region. For bound mitochondria, the position of the mitochondrion was updated by taking a radial outward step with a distance obtained by multiplying the drift velocity by the time step, corresponding to a processivity of ~500 nm, in line with measured values of kinesin processivity (21). Then the simulation was repeated for numerous time steps. The simulation was carried out for 5000 mitochondria; the number of time steps was determined by ensuring that the population of simulated mitochondria settled into a steady-state radial distance distribution. The simulations were also repeated by varying the underlying parameters (mainly PKA_{signal}, k_{bind}, and v_+) to examine the effects of these parameters on the mitochondrial radial density distribution.

RESULTS

To assess the response of mitochondrial positioning to variations in functional demand on MIN6 cells, we established a quantitative framework for measuring mitochondrial location within the cell. To visualize the mitochondria, we

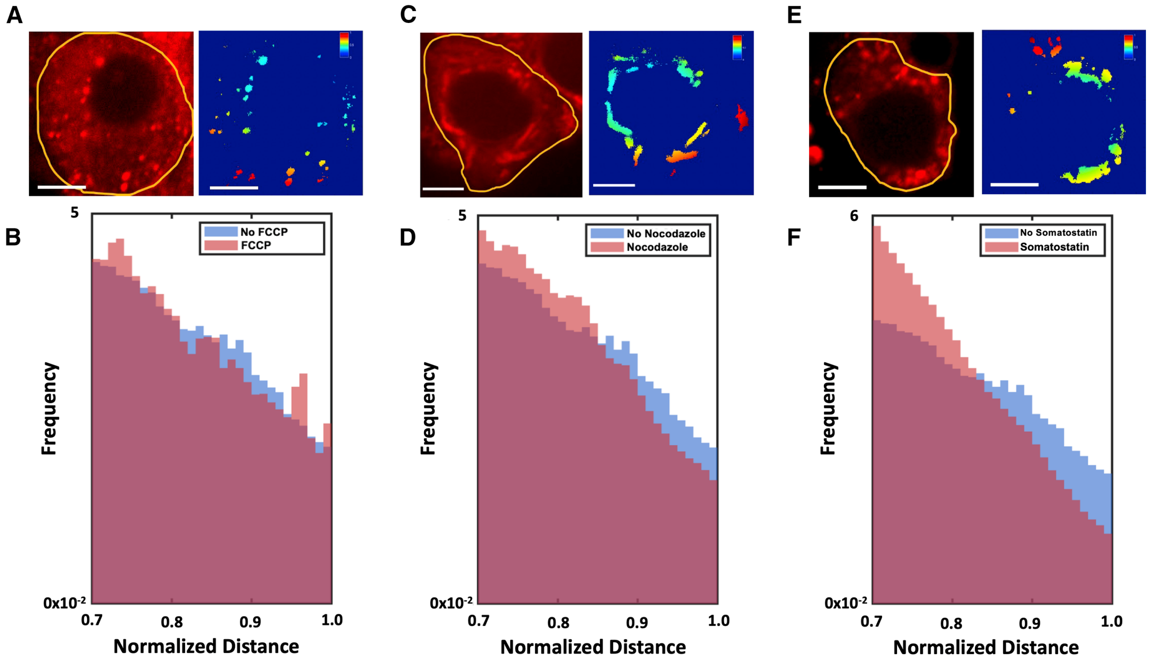

FIGURE 2 Responses of peripheral mitochondrial pixel distance distributions to perturbations in potential regulators of mitochondrial positioning. (*A*) Example micrograph of mitochondrial morphology in response to treatment of MIN6 cells with 10 nM FCCP. Scale bar represents 10 μm. (*B*) Response of mitochondrial pixel distribution to 10 nM FCCP (*blue histogram*, $N = 74$ cells) compared with distribution observed in the absence of FCCP (*red histogram repeated from* Fig. 1 *B*). (*C*) Example micrograph of mitochondrial morphology in response to treatment of MIN6 cells with 10 μM somatostatin. Scale bar represents 10 μm. (*D*) Response of mitochondrial pixel distribution to 10 μM somatostatin (*blue histogram*, $N = 72$ cells) compared with distribution observed in the absence of somatostatin (*red histogram repeated from* Fig. 1 *B*). The two histograms are statistically distinguishable (Kolmogorov-Smirnov test, $p < 1.4 \times 10^{-2}$). (*E*) Example micrograph of mitochondrial morphology in response to treatment of MIN6 cells with 10 μM nocodazole. Scale bar represents 10 μm. (*F*) Response of mitochondrial pixel distribution to 10 μM nocodazole (*blue histogram*, $N = 62$ cells) compared with distribution observed in the absence of nocodazole (*red histogram repeated from* Fig. 1 *B*). The two histograms are statistically distinguishable (Kolmogorov-Smirnov test, $p < 1.1 \times 10^{-7}$).

incubated cells with MitoTracker CMXRos and carried out live-cell spinning disk confocal microscopy (Fig. 1 *A*). We manually identified cell and nuclear boundaries (Fig. 1 *B* and *C*) and then preprocessed the images using a combination of Gaussian and Laplacian filters to remove high-frequency noise in the image. The denoised images were thresholded to segment mitochondrial pixels from the cellular background (Fig. 1 *D*; materials and methods); we verified that our results are relatively insensitive to the detailed choice of thresholding and Gaussian blur parameters (Fig. S1). Crucially, to avoid having to decide exactly how to define mitochondrial position with a summary statistic derived from the segmented pixels, such as the position of the centroid of a particular mitochondrion, we instead measured the distribution of distances of all identified single mitochondrial pixels from the center of the nucleus (Fig. 1 *E* and *F*). A pixel-level description of mitochondrial position allows us to focus on subcellular mitochondrial positioning independent of any potentially confounding effects from mitochondrial fission or fusion, which would not directly influence the location of mitochondrial pixels but would alter the calculated centroid position of individual mitochondria. For each cell, we normalized the distance of a given mitochondrial pixel by the maximum distance recorded between any mitochondrial pixel and the nucleus in that particular cell; we note that the mitochondrial pixel that displayed the maximum distance in each cell coincided with the cell periphery. The 1D coordinate system set up this way mitigates cell-to-cell variability in factors such as cell size, nucleus size, total mitochondrial mass, and the random of adherence to a glass-bottom dish. The relative position of a given mitochondrial pixel is thus also comparable from one cell to another: each pixel has a normalized distance value of at most 1.

With this analysis, we were able to visualize pixel-level histograms of how nuclear-proximal versus membrane-proximal mitochondrial material is organized in space. We repeated these measurements in cells exposed to either low (2 mM) or high (25 mM) glucose concentrations. Although the bulk of the pixel distributions do not show large differences between the two histograms (Fig. S2), we observe a striking difference in the distributions at the peripheral edge of the cells (Fig. 1 *G*; materials and methods). Specifically, in 25 mM glucose, MIN6 cells show a preference for peripheral mitochondria when exposed to 25 mM glucose compared with 2 mM glucose (Fig. 1 *G*). We note no significant differences in average number of mitochondria per cell, average mitochondrial

size per cell, nor average cell size or perimeter (Figs. S3 and S4, materials and methods).

Having detected a difference between the levels of peripheral mitochondria as a function of glucose, we sought to uncover some of the mechanistic underpinnings of this pattern. Inspired by our hypothesis that mitochondria are positioned to help the cell sense and respond to altered extracellular glucose levels, processes that involve interactions at the cell periphery, we tested three distinct factors that could drive spatial repositioning of the mitochondria: the generation of ATP in mitochondria, the increase of intracellular cAMP levels resulting from glucose stimulation, and the presence of a microtubule network along which mitochondria could traffic en route to the cell periphery.

To test the effects of ablating mitochondrial ATP production on mitochondrial position, we downregulated mitochondrial ATP production by treating cells cultured in 25mM glucose to the uncoupling agent FCCP. FCCP is a protonophore that acts to diminish the proton gradient across the mitochondrial inner membrane; as the proton efflux through ATP synthase ultimately drives ATP synthesis, FCCP ultimately acts to reduce ATP production. We verified that FCCP treatment still allowed reliable visualization of mitochondria using MitoTracker CMXRos (Fig. S5, Ref (22)). Interestingly, although we observe significant changes to mitochondrial morphology upon addition of 10 nM FCCP (Fig. 2 *A*), the fraction of mitochondrial pixels proximal to the edge of the cell does not change appreciably compared with when no FCCP is added, especially on the scale of the difference when varying glucose (Figs. 2 *B* and S6 *A*), nor do average mitochondria or number of mitochondria per cell (Fig. S7).

Next, to establish a role for active traffic in establishing glucose-stimulated trafficking of mitochondria to the cellular periphery, we examined the mitochondrial pixel distribution in cells whose microtubule networks were disrupted (23). To effect this disruption, we treated the cells with 10 μM nocodazole, a microtubule destabilizer that fragments the microtubule network on which mitochondria would be transported by the motor proteins kinesin and dynein. We used the dye Spy650-Tubulin to visualize the microtubule network and verify that the network was disrupted upon treatment with nocodazole (Fig. S8, materials and methods). As with other treatments, we observe no gross changes in mitochondrial morphology (Fig. 2 *C*) or average mitochondrial number or size per cell (Fig. S9). With the microtubules depolymerized the histograms show a qualitatively similar response to cells grown in low glucose: we observe a depletion of the mitochondrial radial pixel density function in the peripheral region of the cell. (Figs. 2 *D* and S6 *B*), consistent with the idea that mitochondrial peripheral localization is dependent on an intact microtubule network for anterograde transport.

Finally, to investigate mechanism underlying the signaling process that drives asymmetric mitochondrial traffic along the microtubule network toward the cell periphery as a function of glucose, we targeted the cAMP signaling pathway, a key downstream effector of mitochondrial ATP production (24,25) on mitochondrial positioning. Glucose, for example, has been shown to drive an increase in cAMP levels, which in turn increases kinesin motor protein association and thus could play a role in glucose-dependent mitochondrial positioning. To this end, we incubated our cells with the paracrine inhibitor somatostatin (26). Somatostatin activates an inhibitory G-protein-coupled receptor on beta cells and reduces insulin secretion, at least in part by reducing cAMP levels. Reduced cAMP levels have been implicated in reduced trafficking; for example, it has been shown in neurons that the cAMP/PKA pathway upregulates kinesin activity and thus can play a major role in regulating mitochondrial anterograde transport (20). Mitochondrial morphology as evaluated by visual inspection (Fig. 2 *E*) and average number and size of mitochondria per cell (Fig. S10) does not appreciably change between cells treated and untreated with somatostatin on the timescale we measure. The mitochondrial histograms in response to 10 μM somatostatin reveal a significant decrease in the density of mitochondrial pixels at the most peripheral region of the cells even in the presence of 25 mM glucose (Figs. 2 *F* and S6 *C*), consistent with the idea that glucose-stimulated upregulation of intracellular calcium and in turn the cAMP/PKA pathway could increase mitochondrial anterograde transport and peripheral mitochondrial density.

Our experimental results suggest a minimal model in which mitochondrial interactions with the microtubule network and the cAMP signal transduction pathway play a crucial role in establishing mitochondrial positioning within the cell. To describe the mechanisms governing this dynamic localization, we developed a stochastic computational model to simulate mitochondrial movement and test whether it could qualitatively recreate the shape of the mitochondrial pixel radial density distribution and observed peripheral accumulation.

This model (Fig. 3 *A*) explores the dynamic distribution of mitochondria among two states—free in the cytoplasm or bound to microtubules—based on their binding and unbinding kinetics. The model operates in a circular disk spatial domain whose coordinates represent the percentage of the radial distance between the center and periphery of the modeled cell, thus simplifying the cell's geometry to focus on central-to-peripheral movement. This circular disk geometry reduces computational complexity while capturing the core dynamics of mitochondrial movement. Mitochondria are initially positioned randomly and then undergo a random walk. Reflecting boundary conditions at the perimeter of the circular region constrain mitochondrial positions within the domain, mimicking the physical barrier of the cell membrane.

The model's core feature is its representation of cAMP/PKA/Ca2+-dependent transport along microtubules,

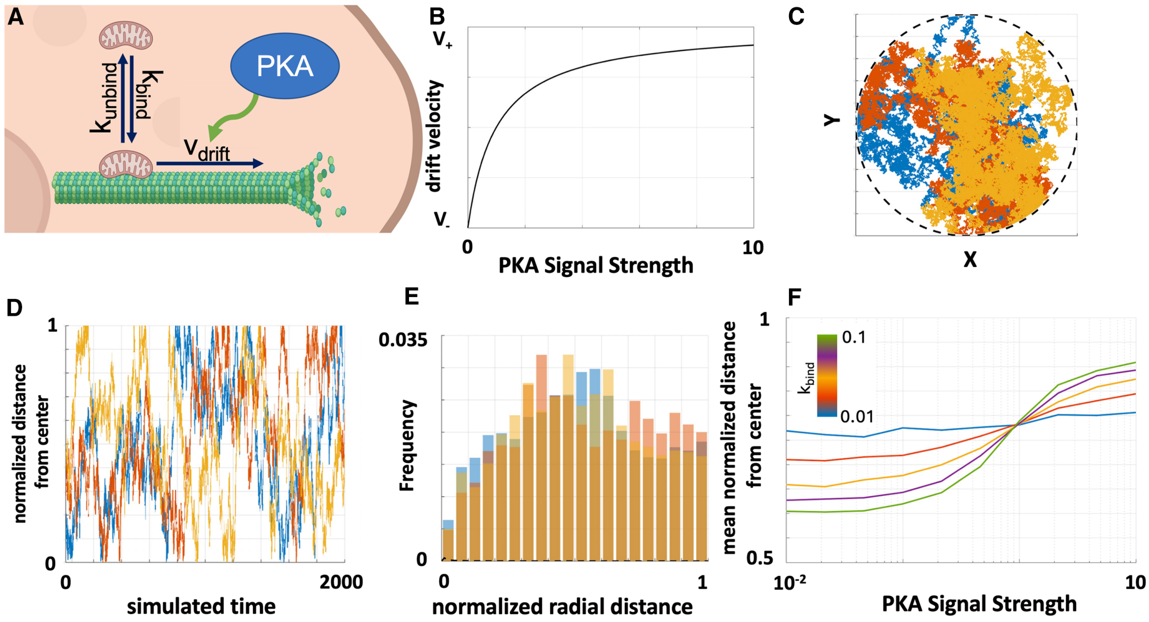

FIGURE 3 Stochastic model of PKA-regulated mitochondrial transport along microtubules. (*A*) Schematic depicting mathematical model of mitochondrial interactions with microtubules. (*B*) Drift velocity as a function of PKA signal strength. (*C* and *D*) Three representative trajectories of simulated mitochondria using our biased random walk simulation. (*C*) tracks the position of the mitochondria within the 2D circular disk geometry they are confined to, and (*D*) shows the time trajectory of the radial distance from the center of the region to each simulated mitochondria. (*E*) Histograms of radial distance of simulated mitochondria from the center of the region. Parameters used in (*C*)–(*E*): $k_{bind} = 0.025$, $PKA_{signal} = 0.7$, $v_+ = v_- = 0.4$. (*F*) Mean mitochondrial radial distance as a function of PKA signal strength and k_{bind} varied as indicated in the colorbar and k_{unbind} held constant.

designed to capture the glucose-induced peripheral trafficking observed in MIN6 cells (Fig. 3 *A*). Mitochondria transition stochastically between free and microtubule-bound states. Free mitochondria undergo a random walk with no preferred direction; though it is worth noting that random walks in circular disk geometries have an inherent bias toward the periphery since the available area for random walkers to explore is proportional to the radial distance from the center of the circle. To model the effects of glucose stimulation on transport along microtubules, we recall that glucose ultimately induces Ca^{2+} influx into the cell, which in turn upregulates PKA signaling (denoted as PKA_{signal}) and consequently kinesin activity and anterograde transport. Microtubule-bound mitochondria can also undergo retrograde transport through the activity of dynein (27). With these kinetics governing the mitochondrial motion, we can define the drift velocity (Fig. 3 *B*) of microtubule-bound mitochondria as

$$v_{drift} = \left(\frac{PKA_{signal}}{1 + PKA_{signal}}\right)v_+ - \left(\frac{1}{1 + PKA_{signal}}\right)v_- \quad (1)$$

Here, PKA_{signal} reflects the signal strength of the cAMP/PKA/Ca^{2+} signaling pathway, and we assume that the PKA pathway cannot drive arbitrarily high velocities and thus will saturate, and v_+ and v_- are parameters characterizing the speed of anterograde and retrograde motion along the microtubules, respectively. We carry out simulations of the 2D mitochondrial random walkers for sufficient time for each walker to explore the entire circular geometry (Fig. 3 *C*), collect the position, and compute the radial distance from the center of the circle of each mitochondrion in the simulation (Fig. 3 *D*), and we thus obtain steady-state mitochondrial radial distance distributions that can be compared with experiment (Fig. 3 *E*). Although we focus on the peripheral edge of the radial distribution, as in the case of our experimental results, we do note that the simulation qualitatively captures the shape of the entire mitochondrial pixel radial density distribution, including the occurrence of the peak of the distribution in a region in between the center and peripheral edge of the cell. Intuitively, we expect this is due to an inherent bias of 2D random walks toward the periphery when the walk takes place in a circular disk geometry and retrograde transport along the microtubules back toward the center of the cell, competing and leading to a buildup of density in the region between the two extremes. Indeed, parameter sensitivity analysis suggests that in the model a peak in the mitochondrial normalized radial distance distribution only occurs near the midpoint between the center and peripheral edge of the cell if $v_{drift} < 0$ (Fig. S11).

To gain an intuitive understanding of the model, we co-varied the PKA_{signal} and k_{bind} parameters. We computed the mean mitochondrial position over each set of simulations for given parameter values and plotted these mean positions as a function of PKA_{signal} and k_{bind}. At very low binding (Fig. 3 *F*, blue curve) we observe that the mitochondrial position remains relatively unaffected no matter the PKA_{signal} value. For appreciable binding of mitochondria

for microtubules, we observe an amplifying-type behavior. An increase in k_{bind} leads to increased peripheral mitochondria when PKA_{signal} is sufficient for $v_{drift} > 0$. Interestingly, we see that if PKA_{signal} is low enough that $v_{drift} < 0$, we see that increasing k_{bind} leads to a reduced mean mitochondrial position. Intuitively, increased binding to the microtubule will amplify the effect of the drift velocity the mitochondria experience while trafficking along microtubules.

Least squares fitting of the simulation results to the experimental mitochondrial pixel normalized radial density distribution suggests that the principal change between the 25 mM and 2 mM glucose distributions is a lower PKA_{signal} value (Fig. S12), though it should be noted that the fit to the 2 mM glucose distribution was improved further with a reduction in the k_{bind} parameter value and the reducing the step size compared with 25 mM glucose (which effectively reduces the diffusion constant of the mitochondria). As expected, the mitochondrial pixel normalized radial distance distributions from somatostatin-treated cells are best fit with a lower PKA_{signal} parameter value. Interestingly, nocodazole treatment in principle could effectively appear in the model as either reducing binding of mitochondria to microtubules or affecting the rate of mitochondrial transport along microtubules. The distributions from nocodazole-treated cells suggest that the shift in the mitochondrial pixel distribution is best attributed to a lowered v_+ rather than a lowered k_{bind}. We interpret this model behavior being a result of the observation that for a peak of the mitochondrial radial distance distribution to be near the middle of the cell, as is the case for the cells treated with nocodazole, $v_{drift} < 0$. But if $v_{drift} < 0$, then if nocodazole decreased k_{bind} the model would expect increased density of mitochondria toward the periphery of the cell, which is not what we observe. Instead, if nocodazole results in a reduction in v_+, we can better explain our data (Fig. S12). It is important to stress, however, that although overlaying the computational simulations with experimental data and comparing their shapes is useful, strictly speaking the comparison is not apples to apples: the experimental data do not consist of point-like mitochondria that generate the distributions we observe, unlike the case of the 2D simulation. Overall, the simulation results are consistent with a model in which calcium influx resulting from glucose stimulation that upregulates PKA activity could result in upregulated anterograde mitochondrial traffic toward the cell periphery along microtubules.

DISCUSSION

Mitochondria produce ATP through oxidative phosphorylation, and careful work has drawn links between mitochondrial-specific geometric properties and the rate at which mitochondria generate ATP (28,29),. Polarized structures, such as neurons, muscle cells, and heart cells, are known to have mitochondrial spatial distribution repositioned, where in these cases the repositioning is hypothesized to metabolically support regions of high energy demand. These cells share the common feature of signal transduction, with a clear need for temporal and spatial efficiency in order to communicate with neighboring cells. Whether these same principles play out in cell types whose morphologies are comparatively less polarized is unclear. We sought to use the pancreatic beta cell-like MIN6 cells as a model system to explore the response of mitochondrial position to environmental stimulus, especially glucose stimulation.

We observed that in conditions of comparatively high energy metabolism, namely high glucose, there was a significant difference in the mitochondrial density at the furthest edges of these cells. This shift in mitochondrial density appears to be primarily dependent on glucose levels regulating the level of cAMP/PKA/Ca^{2+} signaling to promote anterograde traffic of mitochondria along an intact microtubule network. We were able to qualitatively recapitulate our experimental patterns in a mathematical model that described a biased random walk for mitochondria whose drift velocity along the microtubule is proportional to $Ca^{2+/}$PKA signaling.

Although speculative, it is possible that this peripheral mitochondrial localization and morphological changes that possibly assist this transport may help ensure mitochondrial energy production supports insulin secretion (29,30), as localized energy may be necessary for efficient exocytosis of insulin granules. Mitochondrial function extends beyond ATP production; in particular, mitochondria are major regulators of intracellular calcium levels, which play a major role in insulin secretion (18,19), and our somatostatin results suggest that cAMP levels, and thus calcium, could also help regulate mitochondrial position. In the broader context of the interplay between mitochondria and the sensing and response to extracellular glucose levels in beta cells, we note that insulin granules exhibit dissimilar dynamics to what we infer mitochondria obey from our analysis. It was recently shown, for example, that destabilizing the microtubule network via nocodazole treatment inhibits the rate at which insulin granules are withdrawn from the cell periphery (31), unlike the pattern we observe in mitochondria. Indeed, numerous studies have shown that disruption of the actin cytoskeleton, both in the bulk of the cell but especially with disassembly of cortical actin, enhances GSIS (32–35). Although these results have been interpreted as largely a consequence of increased access for insulin granules to sites of exocytosis, it is possible that the mitochondrial peripheral positioning we observe also plays a role. In the language of our mathematical model, the glucose-dependent mitochondrial peripheral positioning could help buffer membrane-proximal calcium levels to regulate insulin secretion, rather than necessarily only serving as a local ATP source. This picture is consistent with the correlation we observe between mitochondrial peripheral positioning to conditions that result in higher insulin secretion.

From the perspective of the cell and its mitochondria, one potential benefit to mitochondrial positioning is the flexibility it affords to the cell in terms of allowing individual mitochondria within the cell to perform the wide variety of biochemical tasks assigned to them. Elevated mitochondrial fusion, for example, globally links mitochondria together into a networked structure well suited to supplying the entirely of the cell with ATP during periods of elevated cellular proliferation (36), but potentially at the cost of homogenizing the mitochondrial compartment, rendering it unable to perform otherwise incompatible biochemical processes simultaneously (10). MIN6 cells, and their pancreatic beta cell counterparts, however, need to balance the calcium and energetic maintenance functions that mitochondria provide to the cell with the increased demands placed on them by glucose stimulation. Mitochondrial positioning in cells such as MIN6 and their pancreatic beta cell counterparts, however, could allow the cell to not be required to extensively remodel mitochondrial composition to elevate ATP production rates needed to boost insulin secretion, but simply to capture them at sites of increased demand to elevate local ATP concentrations.

Finally, although our results and analysis have been focused on MIN6 cells, we note that the phenomenon of spatially inhomogeneous energy demands on cells upon external or internal cues is a widespread phenomenon. From the highly demanding secretory activity of B cells to the epithelia lining the gut, we suggest that mitochondrial positioning may play an important role in matching local energy supply with energy demand in a wide variety of cell biological contexts.

DATA AND CODE AVAILABILITY

All raw image files and analysis code can be located using the following link: https://doi.org/10.5281/zenodo.17545789.

ACKNOWLEDGMENTS

We thank A. Ustione for assistance with microscopy and members of the Piston and Mukherji groups for discussions and critical evaluation of the manuscript. This work was supported by the Washington University Imaging, Modeling and Engineering of Diabetic Tissues Training Grant T32DK108742 (to L.P.), R01DK123301 (to D.W.P.), and R35GM142704 (to S.M.).

AUTHOR CONTRIBUTIONS

L.P., D.W.P., and S.M. conceived the project and designed the experiments. L.P. carried out the experiments with assistance from X.W.N. and M.M. L.P. and S.M. designed the model. L.P., D.W.P., and S.M. wrote the manuscript and acquired funding.

DECLARATION OF INTERESTS

The authors declare no competing interests.

SUPPORTING MATERIAL

Supporting material can be found online at https://doi.org/10.1016/j.bpj.2025.11.018.

REFERENCES

1. Friedman, J. R., and J. Nunnari. 2014. Mitochondrial form and function. *Nature.* 505:335–343.
2. Nunnari, J., and A. Suomalainen. 2012. Mitochondria: in sickness and in health. *Cell.* 148:1145–1159.
3. Youle, R. J., and A. M. van der Bliek. 2012. Mitochondrial fission, fusion, and stress. *Science.* 337:1062–1065.
4. Adebayo, M., S. Singh, …, S. Dasgupta. 2021. Mitochondrial fusion and fission: The fine-tune balance for cellular homeostasis. *FASEB J.* 35:e21620.
5. van der Bliek, A. M., Q. Shen, and S. Kawajiri. 2013. Mechanisms of mitochondrial fission and fusion. *Cold Spring Harbor Perspect. Biol.* 5:a011072.
6. Mishra, P., and D. C. Chan. 2016. Metabolic regulation of mitochondrial dynamics. *J. Cell Biol.* 212:379–387.
7. Lin, M. T., and M. F. Beal. 2006. Mitochondrial dysfunction and oxidative stress in neurodegenerative diseases. *Nature.* 443:787–795.
8. Pernas, L., and L. Scorrano. 2016. Mito-morphosis: mitochondrial fusion, fission, and cristae remodeling as key mediators of cellular function. *Annu. Rev. Physiol.* 78:505–531.
9. Chen, H., A. Chomyn, and D. C. Chan. 2005. Disruption of fusion results in mitochondrial heterogeneity and dysfunction. *J. Biol. Chem.* 280:26185–26192.
10. Ryu, K. W., T. S. Fung, …, C. B. Thompson. 2024. Cellular ATP demand creates metabolically distinct subpopulations of mitochondria. *Nature.* 635:746–754.
11. Kraft, L. M., and L. L. Lackner. 2018. Mitochondrial anchors: Positioning mitochondria and more. *Biochem. Biophys. Res. Commun.* 500:2–8.
12. Yi, M., D. Weaver, and G. Hajnóczky. 2004. Control of mitochondrial motility and distribution by the calcium signal: a homeostatic circuit. *J. Cell Biol.* 167:661–672.
13. Sheng, Z. H., and Q. Cai. 2012. Mitochondrial transport in neurons: impact on synaptic homeostasis and neurodegeneration. *Nat. Rev. Neurosci.* 13:77–93.
14. Hollenbeck, P. J., and W. M. Saxton. 2005. The axonal transport of mitochondria. *J. Cell Sci.* 118:5411–5419.
15. Agrawal, A., G. Pekkurnaz, and E. F. Koslover. 2018. Spatial control of neuronal metabolism through glucose-mediated mitochondrial transport regulation. *eLife.* 7:e40986.
16. Smith, M. J., and M. J. H. Simmons. 2013. Mathematical modeling of mitochondrial transport and dynamics in the cell. *Bull. Math. Biol.* 75:1352–1377.
17. Kabra, U. D., and M. Jastroch. 2023. Mitochondrial Dynamics and Insulin Secretion. *Int. J. Mol. Sci.* 24:13782.
18. Wiederkehr, A., and C. B. Wollheim. 2008. Impact of mitochondrial calcium on the coupling of metabolism to insulin secretion in the pancreatic β-cell. *Cell Calcium.* 44:64–76.
19. Trogden, K. P., J. Lee, …, I. Kaverina. 2021. Microtubules regulate pancreatic β-cell heterogeneity via spatiotemporal control of insulin secretion hot spots. *eLife.* 10:e59912.
20. Vagnoni, A., and S. L. Bullock. 2018. A cAMP/PKA/Kinesin-1 Axis Promotes the Axonal Transport of Mitochondria in Aging Drosophila Neurons. *Curr. Biol.* 28:1265–1272.e4.
21. Andreasson, J. O. L., B. Milic, …, S. M. Block. 2015. Examining kinesin processivity within a general gating framework. *eLife.* 4:e07403.
22. Desai, S., S. Grefte, …, W. J. H. Koopman. 2024. Performance of TMRM and Mitotrackers in mitochondrial morphofunctional analysis

of primary human skin fibroblasts. *Biochim. Biophys. Acta Bioenerg.* 1865:149027.

23. Bracey, K. M., G. Gu, and I. Kaverina. 2022. Microtubules in Pancreatic β Cells: Convoluted Roadways Toward Precision. *Front. Cell Dev. Biol.* 10:915206.
24. Tarasov, A. I., F. Semplici, …, G. A. Rutter. 2012. The mitochondrial Ca2+ uniporter MCU is essential for glucose-induced ATP increases in pancreatic beta-cells. *PLoS One.* 7:e39722.
25. Sun, Y., D. Li, …, F. Li. 2022. Both cellular ATP level and ATP hydrolysis free energy determine energetically the calcium oscillation in pancreatic β-cell. *Phys. Rev. Res.* 4:043142.
26. Rorsman, P., and M. O. Huising. 2018. The somatostatin-secreting pancreatic δ-cell in health and disease. *Nat. Rev. Endocrinol.* 14:404–414.
27. Kashina, A., and V. Rodionov. 2005. Intracellular organelle transport: few motors, many signals. *Trends Cell Biol.* 15:396–398.
28. Randall, E. B., M. Hock, …, D. A. Beard. 2021. Quantitative analysis of mitochondrial ATP synthesis. *Math. Biosci.* 340:108646.
29. Garcia, G. C., K. Gupta, …, P. Rangamani. 2023. Mitochondrial morphology governs ATP production rate. *J. Gen. Physiol.* 155:e202213263.
30. Meng, Y. X., G. W. Wilson, …, R. Balczon. 1997. Suppression of the expression of a pancreatic beta-cell form of the kinesin heavy chain by antisense oligonucleotides inhibits insulin secretion from primary cultures of mouse beta-cells. *Endocrinology.* 138:1979–1987.
31. Zhu, X., R. Hu, …, I. Kaverina. 2015. Microtubules Negatively Regulate Insulin Secretion in Pancreatic β Cells. *Dev. Cell.* 34:656–668.
32. Li, G., E. Rungger-Brändle, …, C. B. Wollheim. 1994. Effect of disruption of actin filaments by Clostridium botulinum C2 toxin on insulin secretion in HIT-T15 cells and pancreatic islets. *Mol. Biol. Cell.* 5:1199–1213.
33. Thurmond, D. C., C. Gonelle-Gispert, …, J. E. Pessin. 2003. Glucose-stimulated insulin secretion is coupled to the interaction of actin with the t-SNARE (target membrane soluble N-ethylmaleimide-sensitive factor attachment protein receptor protein) complex. *Mol. Endocrinol.* 17:732–742.
34. Wang, Z., and D. C. Thurmond. 2009. Mechanisms of biphasic insulin-granule exocytosis - roles of the cytoskeleton, small GTPases and SNARE proteins. *J. Cell Sci.* 122:893–903.
35. Kalwat, M. A., and D. C. Thurmond. 2013. Signaling mechanisms of glucose-induced F-actin remodeling in pancreatic islet β cells. *Exp. Mol. Med.* 45:e37.
36. Yao, C. H., R. Wang, …, G. J. Patti. 2019. Mitochondrial fusion supports increased oxidative phosphorylation during cell proliferation. *eLife.* 8:e41351.

Biophysical Journal, Volume *125*

Supplemental information

Mitochondrial position responds to glucose stimulation in a model of the pancreatic beta cell

Luis Perez, Xue Wen Ng, Michael Mohs, David W. Piston, and Shankar Mukherji

Supplementary Figures

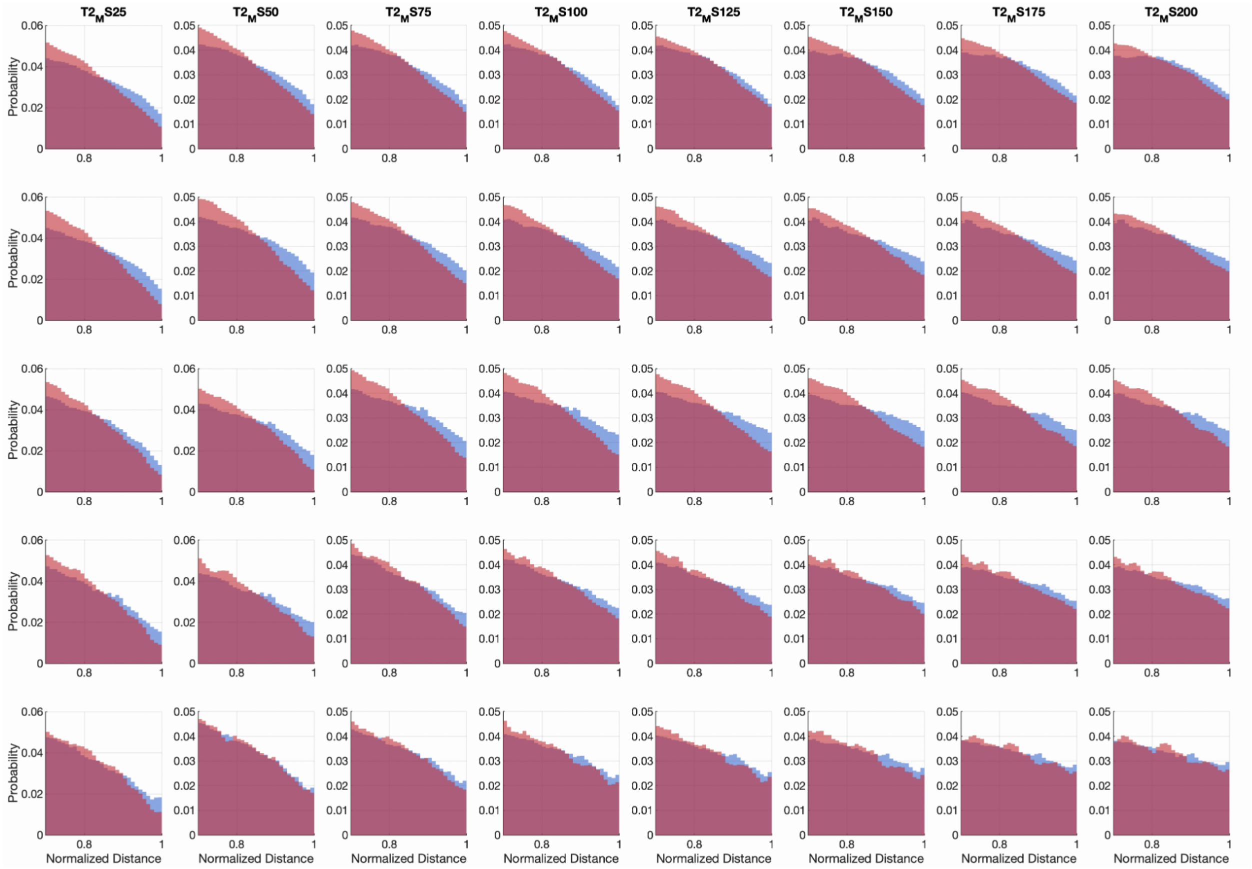

Figure S1. Sensitivity analysis of mitochondrial pixel probability distributions as a function of smoothing and thresholding parameters. Connected component size thresholding parameters are varied across columns between 25 to 200 pixels, fluorescence intensity thresholding parameters are varied across rows from 100-1000.

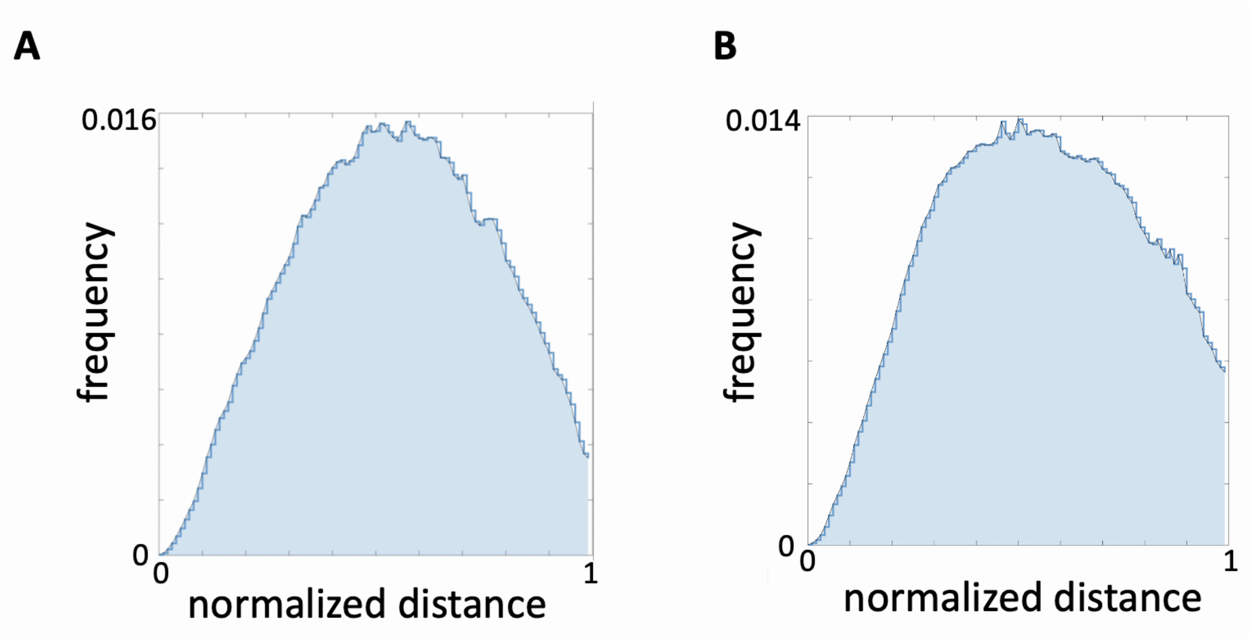

Figure S2: Mitochondrial pixel normalized radial density distributions for cells stimulated with A) 2mM glucose and B) 25mM glucose.

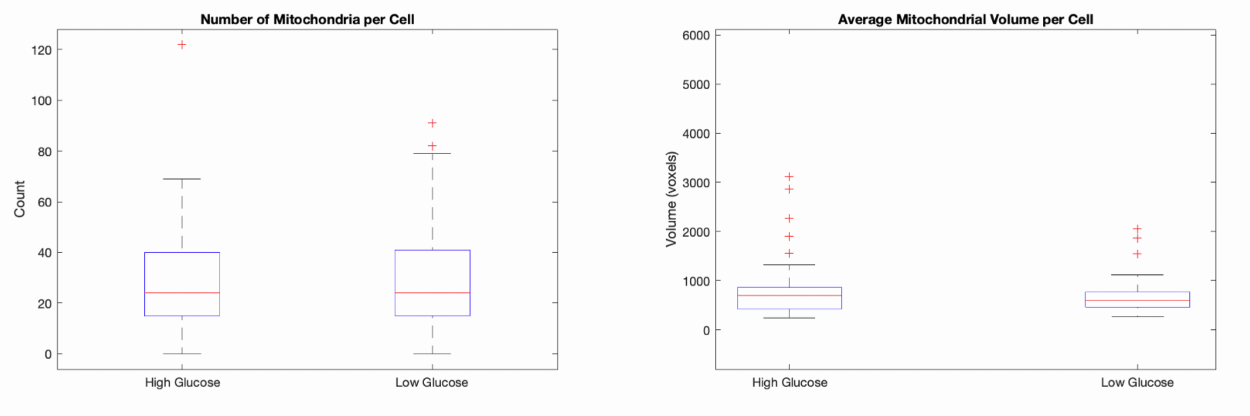

Figure S3. Box and whisker plots showing the median (red line) and 50% interquartile range (blue boxes) of number of mitochondria per cell and average mitochondrial volume per cell for cells cultured in 25mM glucose (high glucose) and 2mM glucose (low glucose)

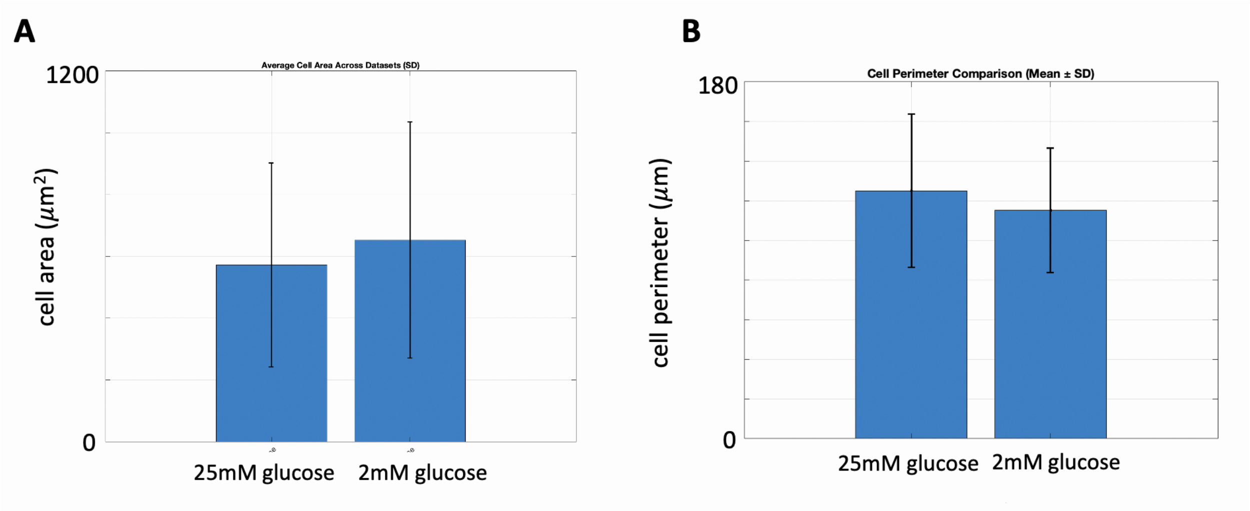

Figure S4: Comparison of cell area (A; p-value = 0.1882, t-test) and cell perimeter (B; p-value = 0.2114, t-test) for cells stimulated with 25mM and 2mM glucose.

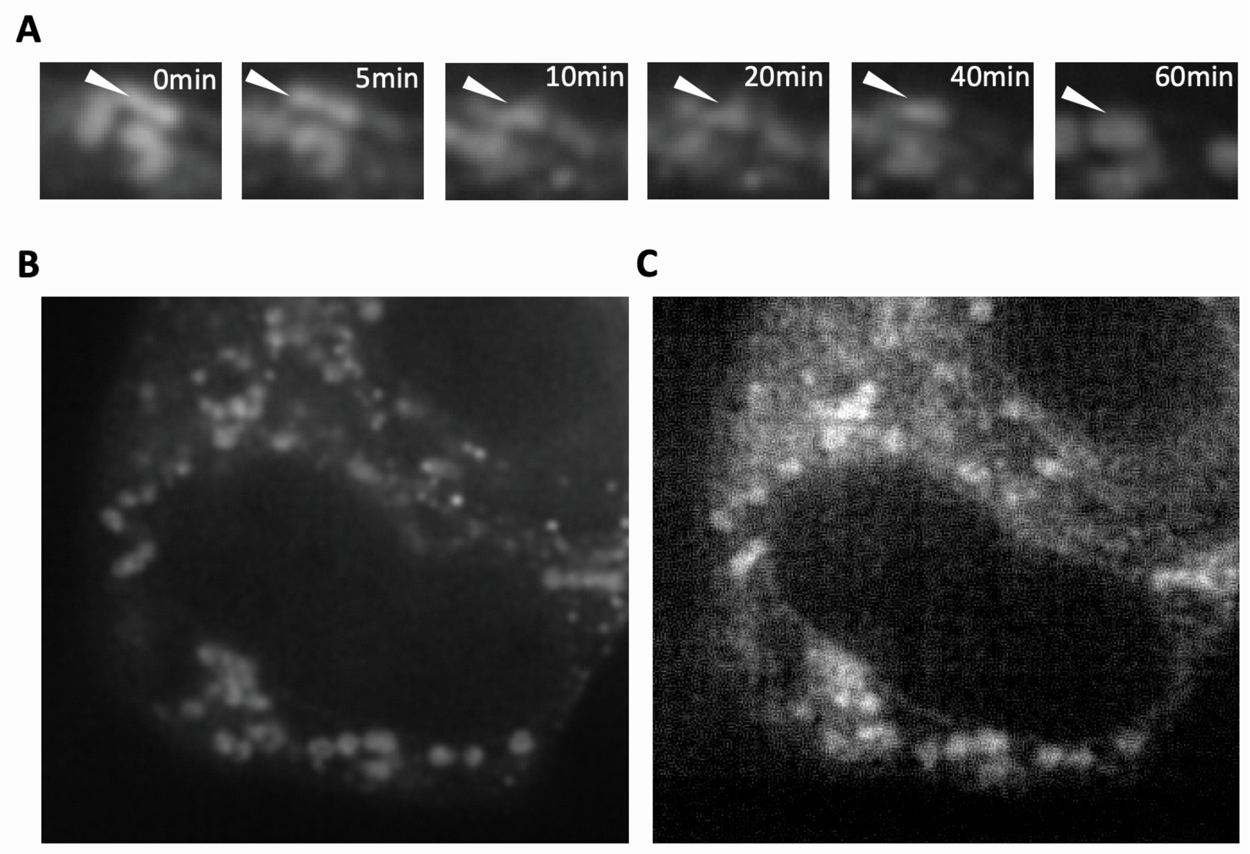

Figure S5: Effect of FCCP on MitoTrackerCMXRos signal. A) Timelapse imaging of single mitochondrion labeled with MitoTrackerCMXRos over a course of 60min. Comparison of fluorescence signal of mitochondrial dually labeled with MitoTrackerCMXRos (B) and MitoTracker Green FM (C).

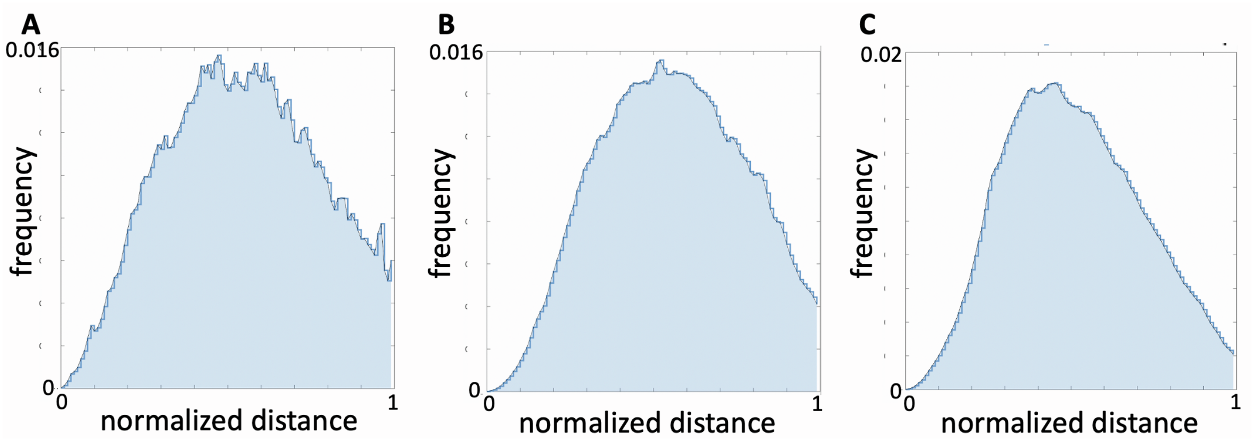

Figure S6: Mitochondrial pixel normalized radial distance density distributions for cells treated with: A) FCCP, B) nocodazole, and C) somatostatin.

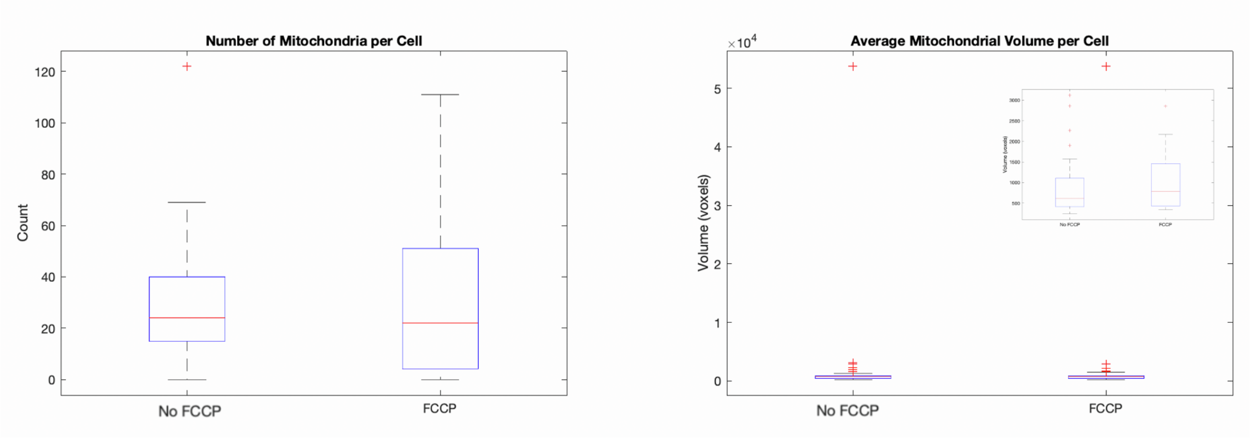

Figure S7. Box and whisker plots showing the median (red line) and 50% interquartile range (blue boxes) of number of mitochondria per cell and average mitochondrial volume per cell in cells cultured in the absence and presence of the uncoupling agent FCCP. Inset in right panel shows box and whisker plots with points with outlier average mitochondrial volume datapoints removed to facilitate comparison between the two populations.

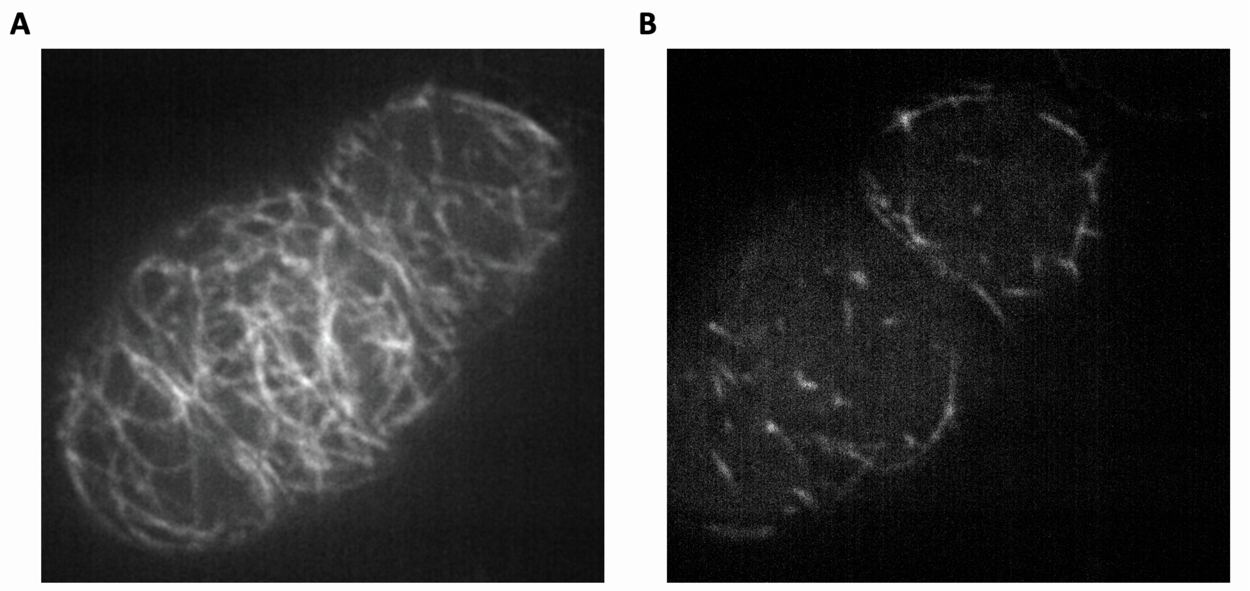

Figure S8: Microtubule networks visualized with Spy650-Tubulin: A) prior to nocodazole treatment, B) following nocodazole treatment.

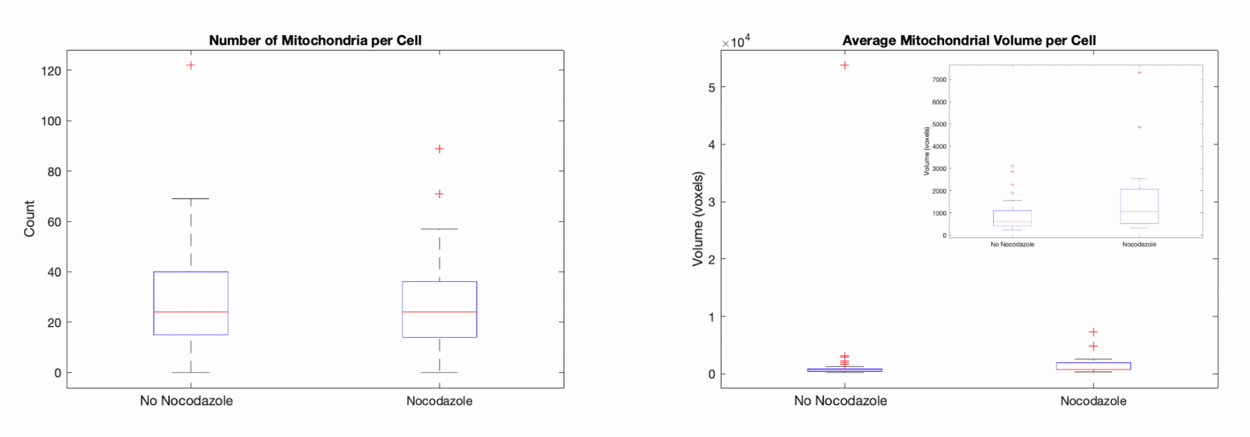

Figure S9. Box and whisker plots showing the median (red line) and 50% interquartile range (blue boxes) of number of mitochondria per cell and average mitochondrial volume per cell in cells cultured in the absence and presence of the microtubule disrupting agent nocodazole. Inset in right panel shows box and whisker plots with points with outlier average mitochondrial volume datapoints removed to facilitate comparison between the two populations.

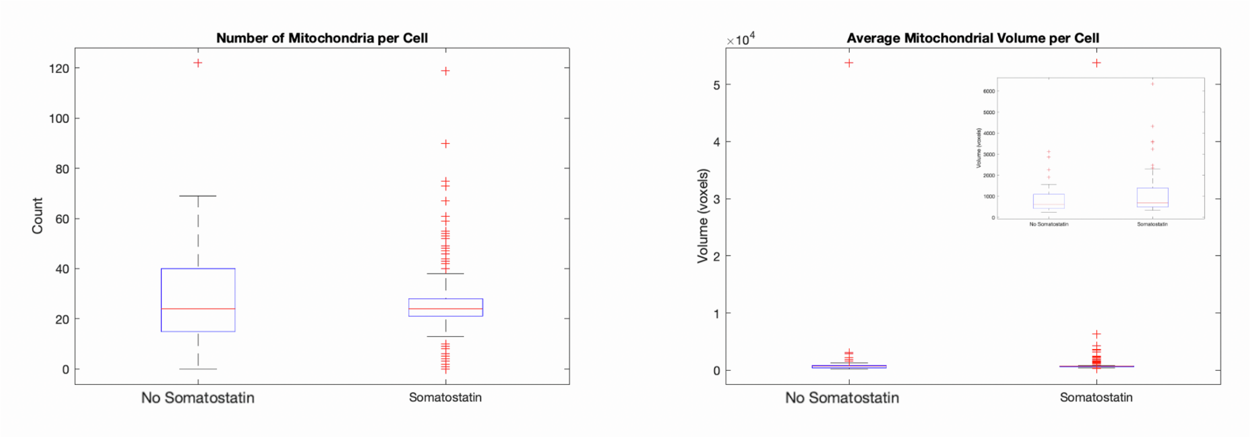

Figure S10. Box and whisker plots showing the median (red line) and 50% interquartile range (blue boxes) of number of mitochondria per cell and average mitochondrial volume per cell in cells cultured in the absence and presence of somatostatin. Inset in right panel shows box and whisker plots with points with outlier average mitochondrial volume datapoints removed to facilitate comparison between the two populations.

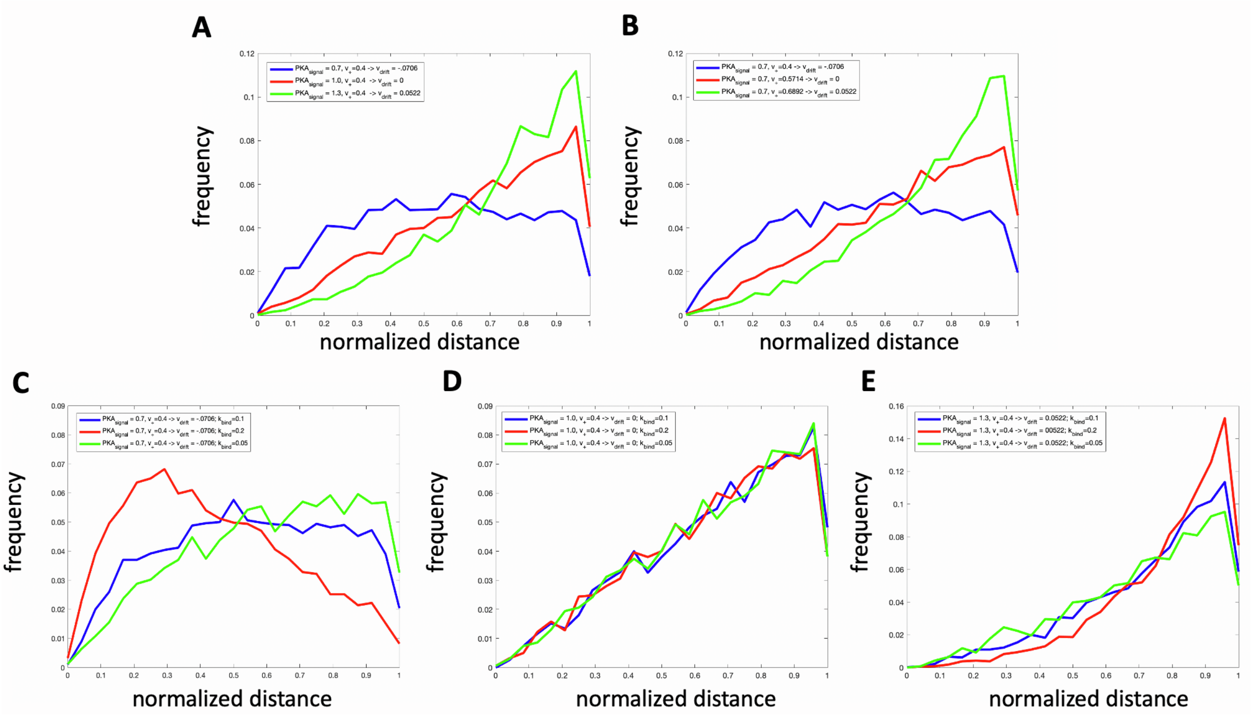

Figure S11: Model parameter sensitivity analysis as plotted in mitochondrial normalized radial distance distributions from simulations. A) Results of sensitivity analysis changing v_{drift} by changing PKA_{signal} but holding v_+ constant. B) Results of sensitivity analysis changing v_{drift} by changing v_+ but holding PKA_{signal} constant. C) Results of sensitivity analysis changing k_{bind} and keeping $v_{drift} < 0$ holding v_+ and PKA_{signal} constant. D) Results of sensitivity analysis changing k_{bind} and keeping $v_{drift} = 0$ holding v_+ and PKA_{signal} constant. E) Results of sensitivity analysis changing k_{bind} and keeping $v_{drift} > 0$ holding v_+ and PKA_{signal} constant.

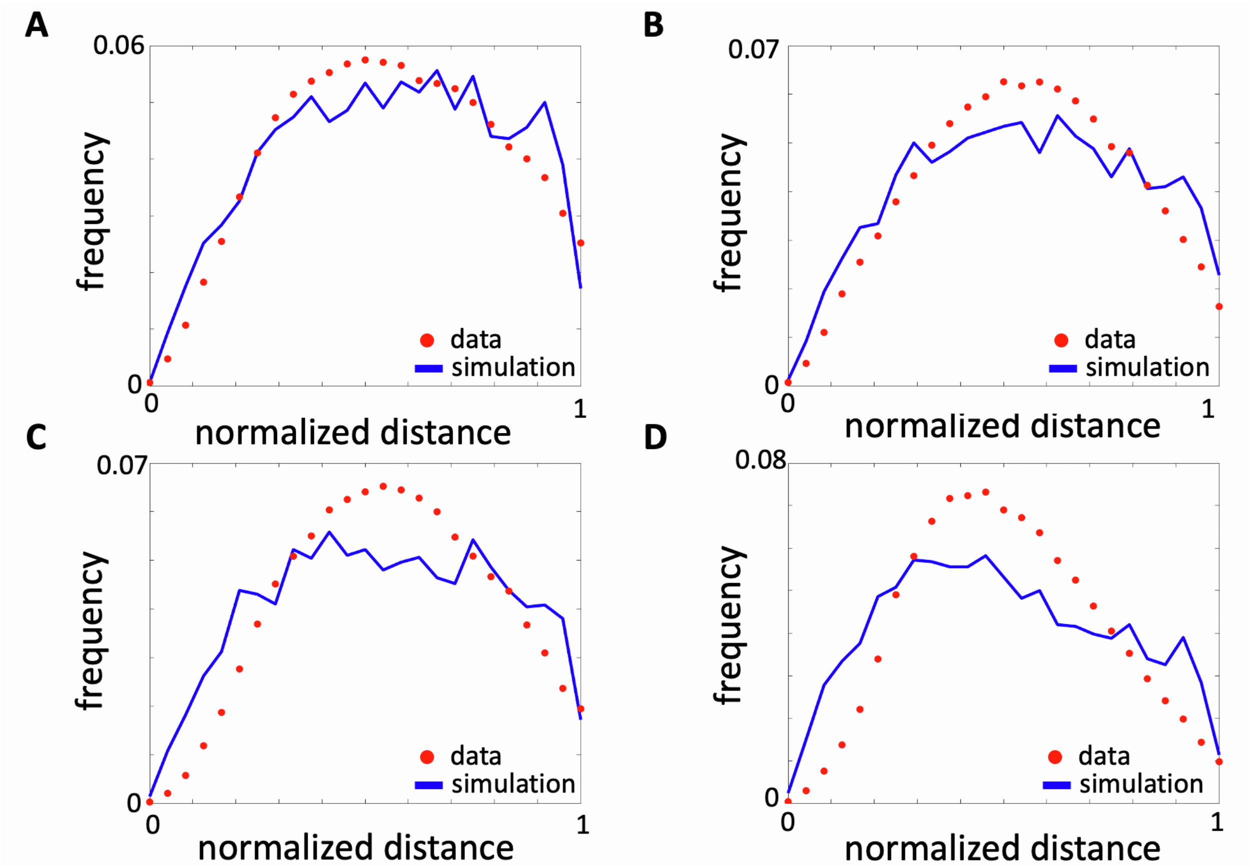

Figure S12: Comparison of random walk model of mitochondria to experimental data of mitochondrial pixel normalized distance distribution. A) 25mM glucose (parameter values k_{bind} = 0.1, PKA_{signal} = 0.7, v_+ = 0.4, v_- = 0.4, step size = 0.2), B) 2mM glucose (parameter values k_{bind} = 0.05, PKA_{signal} = 0.64, v_+ = 0.4, v_- = 0.4, step size = 0.15), C) nocodazole (parameter values k_{bind} = 0.05, PKA_{signal} = 0.7, v_+ = 0.2, v_- = 0.4, step size = 0.2), D) somatostatin. (parameter values k_{bind} = 0.1, PKA_{signal} = 0.6, v_+ = 0.4, v_- = 0.4, step size = 0.2)
